# Additional PfCRT mutations driven by selective pressure for improved fitness can result in the loss of piperaquine resistance and altered *Plasmodium falciparum* physiology

**DOI:** 10.1128/mbio.01832-23

**Published:** 2023-12-07

**Authors:** Laura M. Hagenah, Satish K. Dhingra, Jennifer L. Small-Saunders, Tarrick Qahash, Andreas Willems, Kyra A. Schindler, Gabriel W. Rangel, Eva Gil-Iturbe, Jonathan Kim, Emiliya Akhundova, Tomas Yeo, John Okombo, Filippo Mancia, Matthias Quick, Paul D. Roepe, Manuel Llinás, David A. Fidock

**Affiliations:** 1 Department of Microbiology and Immunology, Columbia University Irving Medical Center, New York, New York, USA; 2 Center for Malaria Therapeutics and Antimicrobial Resistance, Columbia University Irving Medical Center, New York, New York, USA; 3 Division of Infectious Diseases, Department of Medicine, Columbia University Irving Medical Center, New York, New York, USA; 4 Department of Chemistry, Pennsylvania State University, University Park, Pennsylvania, USA; 5 Department of Biochemistry and Molecular Biology and Huck Center for Malaria Research, Pennsylvania State University, University Park, Pennsylvania, USA; 6 Department of Chemistry, Georgetown University, Washington, DC, USA; 7 Department of Biochemistry and Cellular and Molecular Biology, Georgetown University, Washington, DC, USA; 8 Department of Psychiatry, Columbia University Irving Medical Center, New York, New York, USA; 9 Department of Physiology and Cellular Biophysics, Columbia University Irving Medical Center, New York, New York, USA; 10 Area Neuroscience - Molecular Therapeutics, New York State Psychiatric Institute, New York, New York, USA; National Institute of Allergy and Infectious Diseases, Bethesda, Maryland, USA

**Keywords:** *Plasmodium falciparum*, malaria, drug resistance evolution, fitness, *PfCRT*

## Abstract

Malaria elimination efforts in Southeast Asia have been hindered by multidrug-resistant *Plasmodium falciparum*. High-grade resistance to piperaquine (PPQ, used in combination with dihydroartemisinin) is associated with PfCRT mutations that arose in strains expressing the PfCRT Dd2 isoform, which mediates resistance to the related 4-aminoquinoline chloroquine (CQ). The PPQ-resistant PfCRT haplotype Dd2 + F145I mediates the highest level resistance but causes a significant growth defect in intra-erythrocytic parasites. Recently, three separate mutations (F131C, I347T and C258W) have been observed on Dd2 + F145I PfCRT either during extended parasite culture or in Southeast Asian isolates no longer subject to PPQ pressure. Competitive growth assays with *pfcrt*-edited parasites reveal that these compensatory mutations reduce the fitness defect caused by F145I. PPQ survival assays on edited lines show a loss of PPQ resistance in two of the three variants, including the field mutant (C258W). The latter restores CQ resistance. None of these variants alter parasite susceptibility to the first-line partner drug, mefloquine. Utilizing drug transport assays with purified PfCRT isoforms reconstituted into proteoliposomes, we identify differences in mutant PfCRT-mediated transport of PPQ and CQ. Molecular dynamics energy minimization calculations predict that these same mutations cause small but significant conformational changes in PfCRT regions implicated in drug interactions. Metabolomic analyses of isogenic parasite lines reveal differences in hemoglobin-derived peptide accumulation as a hallmark of PfCRT variation. These studies highlight the transient nature of PPQ resistance upon removal of drug pressure and suggest a strategy for employing this drug as part of multiple first-line therapies.

**IMPORTANCE:**

Our study leverages gene editing techniques in *Plasmodium falciparum* asexual blood stage parasites to profile novel mutations in mutant PfCRT, an important mediator of piperaquine resistance, which developed in Southeast Asian field isolates or in parasites cultured for long periods of time. We provide evidence that increased parasite fitness of these lines is the primary driver for the emergence of these PfCRT variants. These mutations differentially impact parasite susceptibility to piperaquine and chloroquine, highlighting the multifaceted effects of single point mutations in this transporter. Molecular features of drug resistance and parasite physiology were examined in depth using proteoliposome-based drug uptake studies and peptidomics, respectively. Energy minimization calculations, showing how these novel mutations might impact the PfCRT structure, suggested a small but significant effect on drug interactions. This study reveals the subtle interplay between antimalarial resistance, parasite fitness, PfCRT structure, and intracellular peptide availability in PfCRT-mediated parasite responses to changing drug selective pressures.

## INTRODUCTION

Morbidity and mortality rates of *Plasmodium falciparum* malaria continue to be exceedingly high, with an estimated 241 million cases and 619,000 deaths in 2021 (1). Artemisinin (ART)-combination therapies (ACTs) serve as the first-line treatment for *P. falciparum* worldwide (2). This treatment consists of a potent yet rapidly eliminated ART derivative that is paired with a longer lasting partner drug, such as mefloquine (MFQ), amodiaquine, or piperaquine (PPQ), to eliminate residual parasites. Unfortunately, a major threat to the efficacy of these treatments is the recent emergence and spread of *P. falciparum* strains that are resistant to one or both ACT components (3).

PPQ has numerous benefits as an ACT partner drug, including its safety profile in children and pregnant women, its potency against chloroquine (CQ)-resistant parasites, and its long half-life (~20–30 days). The pharmacokinetics of PPQ enable post-treatment prophylactic effects, which makes the dihydroartemisinin (DHA)-PPQ combination attractive for chemoprevention treatment in regions of Africa (4). DHA-PPQ has proven to be effective for both seasonal malaria chemoprevention (intermittent administration of full treatment courses to children during the malaria season in areas of increased seasonal transmission) and intermittent preventative treatment (administration of full courses to pregnant women and infants to prevent malaria infection in these high-risk groups) (5). In addition, MFQ paired with DHA-PPQ has been proposed as an effective triple combination therapy to impede the development of multidrug-resistant strains (6). For the DHA-PPQ combination, reports of widespread treatment failure in Southeast (SE) Asia raise concerns about its long-term effectiveness and highlight the need to understand mechanisms of PPQ resistance (7).

PPQ, like the related 4-aminoquinoline drug CQ, is believed to accumulate within the parasite’s digestive vacuole (DV) where it presumably acts upon the hemoglobin (Hb) degradation pathway. Experimental evidence suggests that PPQ binds to toxic free heme monomers released during Hb proteolysis, thereby preventing their incorporation into chemically inert hemozoin crystals (8). Studies have associated PPQ resistance with mutations in the DV membrane-resident protein PfCRT (*P. falciparum* chloroquine resistance transporter), as confirmed by gene editing (9–16). Evidence suggests that these mutations enable the transporter to efflux positively charged PPQ out of its site of action in the DV (17–19), resulting in parasite survival under high drug concentrations. PPQ-resistant (PPQ-R) PfCRT mutations have also been linked to alterations in Hb metabolism, DV swelling, and decreased parasite fitness (12, 15, 16, 20, 21).

In SE Asia, PPQ-R point mutations arose primarily on parasites expressing the CQ-resistant Dd2 PfCRT, which differs from the 3D7 wild-type (WT) isoform by eight mutations (Table 1) (22). Interestingly, PPQ-R mutations often re-sensitize parasites to CQ, despite the presence of the K76T amino acid substitution that is often used to define CQ resistance-associated PfCRT isoforms. PPQ resistance has also been associated with amplification of *plasmepsins II/III* that encode hemoglobinases (23). Nevertheless, mutant PfCRT haplotypes have been shown to mediate high-level PPQ resistance even in parasites with single copies of these plasmepsins (12). PPQ resistance *in vitro* is characterized by atypical bimodal dose-response profiles and >10% of ring-stage parasites surviving a 48-h exposure to 200 nM PPQ, as measured in the PPQ survival assay (PSA) (9). Resistant parasites *in vitro* also exhibit incomplete parasite killing at elevated PPQ concentrations, manifesting in an increased IC_90_ (the drug concentration that inhibits parasite growth by 90%) (12). Of the SE Asian PPQ-R haplotypes, Dd2 + F145I exhibits the highest levels of survival under PPQ treatment (>25% survival at concentrations of 200 nM PPQ) (12).

**TABLE 1:** PfCRT isoforms of edited parasites used in this study^*a*^

PfCRT isoform	Origin	PfCRT amino acid at listed positions											
		74	75	76	131	145	220	258	271	326	347	356	371
Dd2	SE Asia	I	E	T	F	F	S	C	E	S	I	T	I
Dd2 + F145I	Cambodia	I	E	T	F	I	S	C	E	S	I	T	I
Dd2 + F145I + F131C	Lab	I	E	T	C	I	S	C	E	S	I	T	I
Dd2 + F145I + I347T	Lab	I	E	T	F	I	S	C	E	S	T	T	I
Dd2 + F145I + C258W	SE Asia	I	E	T	F	I	S	W	E	S	I	T	I
3D7 (WT)	E Africa	M	N	K	F	F	A	C	Q	N	I	I	R

^*a*^ *pfcrt* was edited in Dd2 parasites using customized zinc finger nucleases to express the PfCRT isoforms listed above (see Fig. S1). The origin of the PfCRT isoform is noted. E, East; SE, Southeast.

In addition to antimalarial susceptibility, parasite fitness influences which strains dominate in the field. In *P. falciparum*, fitness broadly includes the capacity for sexual development and transmission as well as the asexual blood stage (ABS) growth rate. Previous analyses of isogenic parasites expressing variant *pfcrt* alleles have shed light on their ability to influence ABS growth rates and have uncovered important differences regarding their global spread (24, 25). Among the PPQ-R mutants, the highly resistant Dd2 + F145I allele imparts the most considerable growth defect (12). In contrast, other variants, such as Dd2 + T93S and Dd2 + I218F, have increased rapidly in Cambodia, which can be explained by their combination of relatively high-grade resistance and low fitness cost (15).

In 2016, policy guidelines in Cambodia changed first-line treatment from DHA-PPQ to artesunate-MFQ in response to widespread PPQ resistance (26). However, since large amounts of drug were still circulating in this area, PPQ usage was not officially stopped until years later. Due to the large fitness cost that PPQ-R PfCRT mutations impart on the parasite, we hypothesized that the proportion of parasites with these mutations may decrease in the absence of PPQ pressure. A large genome sequencing effort applied to 20,000 parasite isolates across Asia and Africa (MalariaGEN Pf7) identified additional mutations on Dd2 + F145I PfCRT in SE Asia (27). We also observed this phenomenon with *in vitro* Dd2 + F145I parasites cultured for extended periods of time.

Herein, we utilized gene editing to investigate the impact of recently identified secondary PfCRT amino acid substitutions on parasite fitness and PPQ susceptibility. These studies are of particular importance in determining how the parasite landscape can change following the removal of PPQ pressure. Our data reveal that these novel mutations improve the ABS replication rate of Dd2 + F145I parasites, suggesting evolutionary pressure to diminish the fitness cost imposed by this PfCRT mutation in the absence of PPQ usage. These novel haplotypes confer varying susceptibility to PPQ. To probe deeper into the impact of these mutant PfCRT haplotypes and to further inspect any connection between PfCRT structure and function, we present data on drug transport kinetics and peptidomics for purified PfCRT and genetically edited *P. falciparum* strains, respectively. Collectively, our studies reveal a broad effect of PfCRT mutations on parasite physiology, which underscores the instability of these mutations in SE Asia.

## RESULTS

### Additional mutations developed on Dd2 + F145I PfCRT

Following an extended period of continuous ABS culture (>60 days) of our gene-edited Dd2^Dd2+F145I^ line (12), we observed an increased growth rate. By sequencing the *pfcrt* locus, we identified two new mutations, F131C and I347T, which appeared independently in this culture. Clones of each mutant line (Dd2^Dd2+F145I+F131C^ and Dd2^Dd2+F145I+I347T^) were obtained using limiting dilution, and whole-genome sequencing confirmed that single-nucleotide polymorphisms in *pfcrt* were the only major difference between these lines and parental Dd2^Dd2+F145I^. Of note, there was no amplification of the *plasmepsins II/III* locus.

We also focused on the Dd2 + F145I + C258W variant that was reported by MalariaGEN investigators as the most prevalent contemporary PfCRT mutation that had recently emerged in eastern SE Asia in isolates carrying PPQ-R PfCRT mutations. This additional mutation was identified in 16 isolates that all harbored the PfCRT Dd2 + F145I mutations (27).

Using customized gene editing (28), we introduced these mutant alleles into a Dd2 parasite in place of its endogenous *pfcrt* allele (Table 1; Fig. S1). Isogenic parasite lines (referred to as edited or “ed”) were engineered to express the PfCRT isoforms Dd2 + F145I + F131C, Dd2 + F145I + I347T, or Dd2 + F145I + C258W. For the former two, gene editing was used to independently confirm the impact of the F131C or I347T mutations in the *in vitro*-derived lines. All three lines were compared to Dd2^Dd2+F145I^, their isogenic parent. Our control lines were Dd2^Dd2^ and Dd2^3D7^, which express the CQ-resistant isoform Dd2 and the WT drug-sensitive PfCRT isoform 3D7, respectively (12, 28, 29). Both the edited lines and the original clones were assayed for any changes in parasite fitness or drug susceptibility.

### Second-site mutations alleviate the fitness cost imparted by the *pfcrt* Dd2 + F145I allele

**Fig 1:**
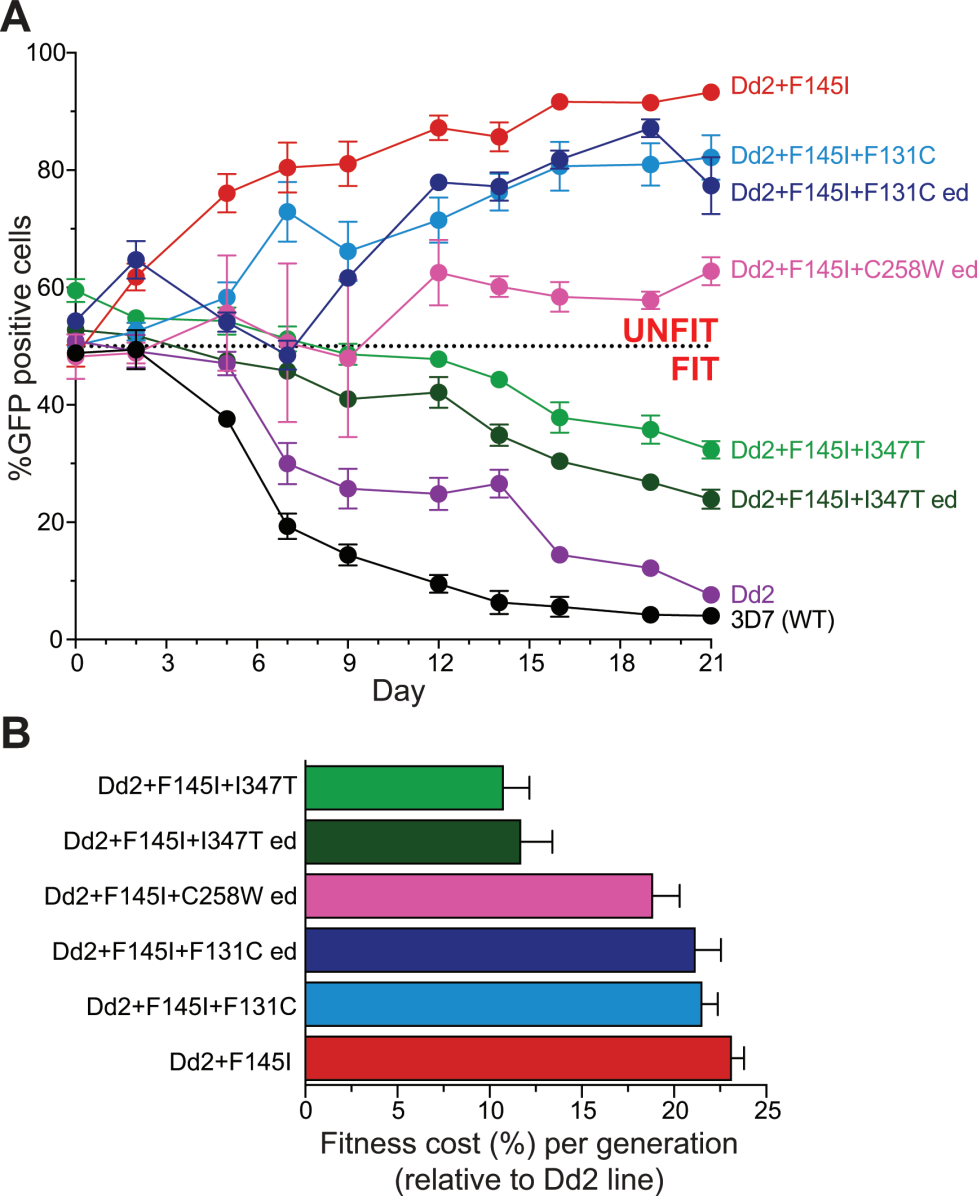
Mutations reduce fitness cost of Dd2 + F145I PfCRT *in vitro*. (**A**) Isogenic *pfcrt*-modified parasite lines, each generated in the Dd2 strain, were co-cultured at an approximately 1:1 ratio with a reference Dd2 line expressing GFP (Dd2-GFP). The labeling indicates the PfCRT isoform expressed by each gene-edited line. The *pfcrt*-edited (ed) lines expressing Dd2 + F145I + F131C, Dd2 + F145I + C258W, or Dd2 + F145I + I347T were generated using customized zinc finger nucleases. Cultures were analyzed by flow cytometry every 2–3 days up to day 21 (10.5 parasite generations), and the mean ± SEM percentages of GFP^+^ parasites were plotted over time. Values above the 50% dashed line indicate that the test line was less fit than Dd2-GFP. (**B**) Mean ± SEM relative fitness cost shown as a percent reduction in parasite growth per 48-h generation is shown, relative to the isogenic Dd2^Dd2^ line. *N* = 2–3; *n* = 2.

To assess the impact of these mutations on ABS parasite growth rates, we used an established flow cytometry-based co-culture assay (12, 15, 24). Parasite cultures were seeded at an approximately 1:1 ratio of a GFP-expressing Dd2 line (Dd2-GFP) and each Dd2 test line (that was GFP negative). Samples were then examined by flow cytometry every 2 to 3 days for 21 days (~10.5 parasite generations) to determine the percentages of GFP^+^ parasites. Dd2^3D7^ and Dd2^Dd2^ were the most fit, outcompeting the Dd2-GFP line within a week, with expression of the 3D7 (WT) isoform resulting in the fastest growth rate. Of the new PfCRT variants, only Dd2^Dd2+F145I+I347T^ showed improved fitness relative to the GFP reporter line (Fig. 1A).

From these data, we extrapolated the change in parasite fitness relative to Dd2^Dd2^ by calculating the percent change in growth rate per 48-h generation for each test line (Fig. 1B). The Dd2^Dd2+F145I^ line was the most unfit, with a 23.1% fitness cost per generation, consistent with prior reports (12, 15, 16). When compared to Dd2^Dd2+F145I^, we observed a modest improvement in fitness in Dd2^Dd2+F145I+F131C^ parasites (calculated at 21.5% and 21.2% cost for the original and edited clone, respectively, relative to Dd2^Dd2^). However, the addition of I347T to Dd2 + F145I PfCRT significantly improved the fitness of both the original mutant parasite and the edited clone (10.8% and 11.7% cost, respectively, relative to Dd2^Dd2^). The Dd2 + F145I + C258W isoform, seen in SE Asian parasites, had a diminished fitness cost of only 18.9% relative to Dd2^Dd2^ (compared to the 23.1% cost of the F145I mutation alone). This suggests that the C258W mutation emerged on a Dd2 +F145I PfCRT background to increase parasite ABS growth rate in the absence of sustained PPQ pressure.

Parasites expressing Dd2 + F145I + F131C, Dd2 + F145I + I347T, or Dd2 + F145I + C258W PfCRT displayed distended DVs in the trophozoite stage, similar to other PPQ-R lines, but the vacuoles in the schizont stage appeared to be smaller compared to those in Dd2^Dd2+F145I^ (Fig. S2). This bloating in the trophozoite and schizont stages is a characteristic feature of PPQ-R lines (10, 12, 29).

### Novel mutant PfCRT alleles increase parasite sensitivity to PPQ albeit to varying degrees

**Fig 2:**
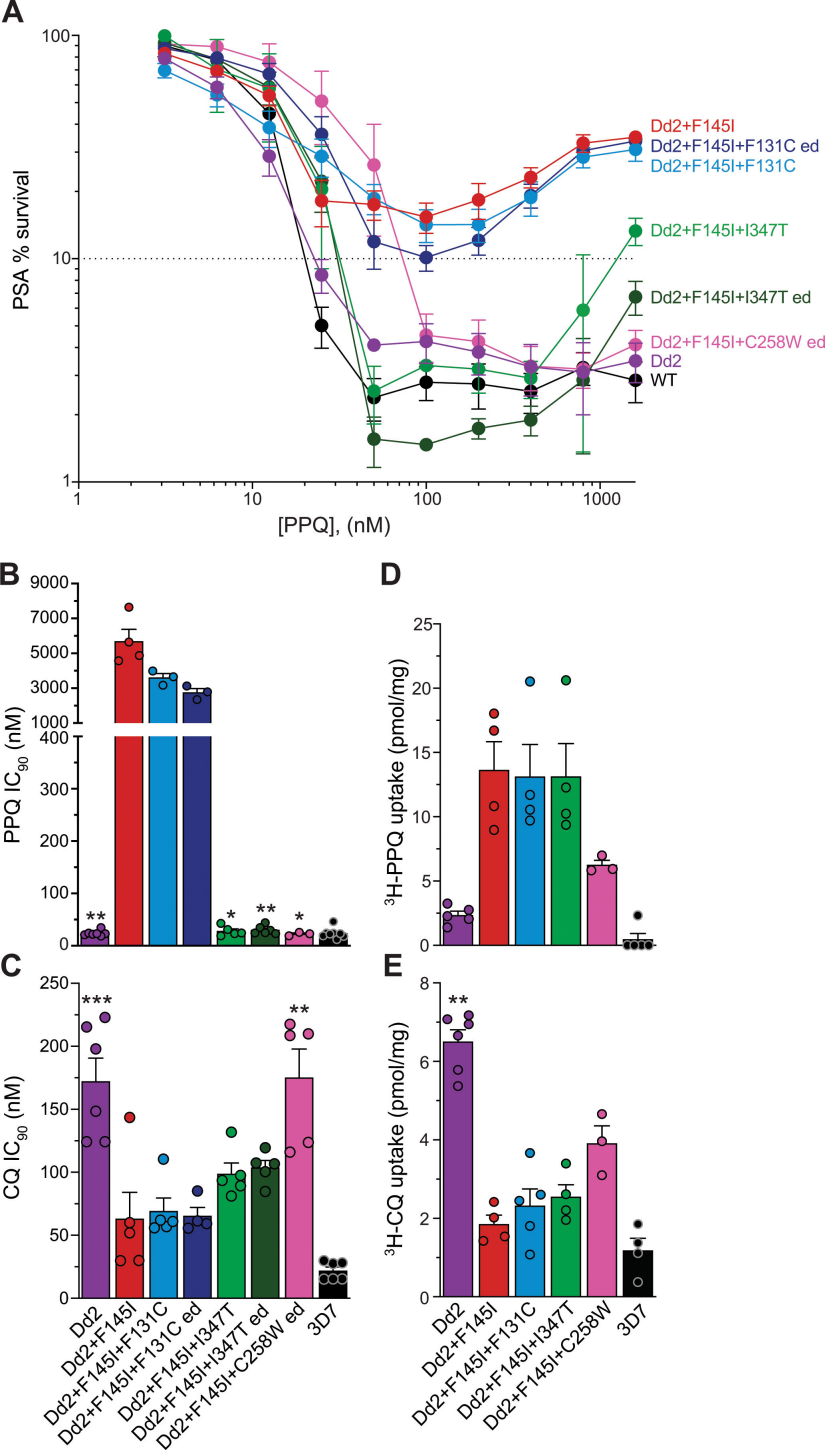
Dd2^Dd2+F145I+C258W^ and Dd2^Dd2+F145I+I347T^ display increased susceptibility to PPQ and altered susceptibility to CQ due to altered drug transport via PfCRT. (**A**) Survival rates of isogenic *pfcrt*-modified lines, each generated in the Dd2 strain, were determined using the *in vitro* PSA (starting with 0- to 6-h rings and treated for 72 h). The 10% cutoff represents a standard 200 nM threshold for PPQ resistance in this assay (30). The labeling indicates the PfCRT isoform expressed by each recombinant line. “ed” refers to lines where the novel PfCRT mutation (F131C, I347T, or C258W) was gene edited into the Dd2^Dd2+F145I^ parasite line. Survival values are shown in Table S1 as means ± SEM. *N* = 7; *n* = 2. (**B and C**) Mean ± SEM IC_90_ values (Table S2) were calculated from 72-h concentration-response assays for (**B**) PPQ and (**C**) CQ. *N =* 4–7; *n* = 2. Significance was determined using Mann-Whitney *U* tests. Comparisons are shown between *pfcrt*-edited lines and their isogenic controls. (**D and E**) Mean ± SEM uptake of 100 nM (**D**) ^3^H-PPQ or (**E**) ^3^H-CQ was measured for 1 min in proteoliposomes containing the indicated PfCRT variants or in control liposomes. Data are means of *n* = 4–6. *P < 0.05; **P < 0.01; ***P < 0.001. (**B–E**) Individual circles indicate values from each independent experiment.

Using the PSA across a range of concentrations (1,600 nM to 3.1 nM PPQ) (12, 15, 30), we tested our edited and original mutant parasite lines for any changes in PPQ susceptibility. The control line Dd2^Dd2+F145I^ showed high levels of survival across a range of high concentrations (32.9%, 18.4%, and 15.4% survival at 800 nM, 200 nM, and 100 nM PPQ, respectively; Fig. 2A; Table S1). The addition of the F131C mutation to Dd2 + F145I PfCRT did not significantly alter PPQ survival rates for either the original mutant (28.5%, 14.2%, and 14.2% at 800 nM, 200 nM, and 100 nM PPQ, respectively) or the edited line (30.5%, 12.1%, and 10.1% at 800 nM, 200 nM, and 100 nM PPQ, respectively; *P* value ≥0.05 using Mann-Whitney *U* tests for both the original and the edited lines as compared to the isogenic Dd2^Dd2+F145I^ line). However, both the I347T and C258W second-site mutations affected PPQ susceptibility. I347T sensitized both the original and the edited clones, which displayed survival rates well below the 10% resistance threshold at 200 nM PPQ (1.7% and 3.2%, respectively). Similarly, C258W parasites became largely re-sensitized, with 4.3% survival at 200 nM PPQ.

We also measured PPQ susceptibility using 72-h dose-response assays with asynchronous parasite cultures. While all tested lines had similar IC_50_ values (Fig. S3A), only Dd2^Dd2+F145I+F131C^ parasites had sustained growth at high concentrations of PPQ. These clones (original and edited) had comparable mean IC_90_ values to Dd2^Dd2+F145I^ (3.6 µM and 2.8 µM compared to 5.6 µM; Fig. 2B; Table S2). These F131C lines displayed an ~3,000 to 5,000-fold increase compared to the Dd2 mean IC_90_ (24 nM). This is consistent with previous observations that PPQ resistance is only apparent at high drug concentrations, underscoring the importance of the PSA to quantify PPQ resistance instead of relying on IC_50_ metrics.

### Second-site mutations result in altered susceptibility to CQ and monodesethyl-CQ

We next assessed the response of these lines to a set of reference antimalarial drugs. Prior studies revealed that the F145I mutation hypersensitizes parasites to CQ and the primary CQ metabolite monodesethyl (md)-CQ, despite arising on the CQ-resistant Dd2 isoform (12). Our data show that the lab-selected Dd2^Dd2+F145I+F131C^ and Dd2^Dd2+F145I+I347T^ lines retained a CQ-susceptible status (Fig. 2C; Table S2). Interestingly, parasites expressing the field isoform Dd2 + F145I + C258W exhibited increased CQ and md-CQ mean IC_90_ values (175 nM and 1.6 µM, respectively) compared to Dd2^Dd2+F145I^ (63 and 317 nM, respectively). The response of Dd2^Dd2+F145I+C258W^ to these drugs did not statistically differ from that of Dd2^Dd2^ (whose CQ and md-CQ mean IC_90_ values were 172 nM and 1.2 µM, respectively; Fig. 2C; Table S2). There was no change in susceptibility to quinine, md-amodiaquine, or MFQ with any of the original or edited lines (Fig. S3C, D, and F). The pyronaridine (PND) mean IC_50_ value of Dd2^Dd2+F145I+C258W^ showed a small (twofold) albeit significant decrease compared to Dd2^Dd2+F145I^ (*P* = 0.029 in Mann-Whitney *U* test; Fig. S3E), suggesting that PfCRT mutations can modulate parasite susceptibility to PND.

### PfCRT-mediated PPQ and CQ uptake correlates with *in vitro* parasite susceptibility

We next sought to probe the impact of these novel mutations on PPQ and CQ transport via PfCRT. Dd2 + F145I + F131C, Dd2 + F145I + I347T, and Dd2 + F145I + C258W isoforms were heterologously expressed in eukaryotic HEK293 cells and purified, along with Dd2 + F145I (PPQ-R, CQ sensitive), Dd2 (PPQ sensitive and CQ resistant), and 3D7 (PPQ and CQ sensitive) as controls (17). As a proxy for transport, we measured uptake of ^3^H-PPQ or ^3^H-CQ by PfCRT-containing proteoliposomes (Fig. 2D and E; Table S3). In this system, an inwardly directed pH gradient (interior alkaline) and electrical transmembrane potential (negative inside) serve as driving forces for PfCRT-mediated drug transport, mimicking the physiological environment under which drug is effluxed out of the parasite DV. Transport studies were performed by adding 100 nM ^3^H-PPQ or ^3^H-CQ to the external medium to initiate the 1-min uptake measurements, which in our assays reflected the maximal transport activity (17).

The highly PPQ-R Dd2 + F145I isoform had the highest PPQ uptake activity, followed closely by Dd2 + F145I + F131C and Dd2 + F145I + I347T (Fig. 2D). These similar levels of PPQ uptake contrast with differences in the PPQ susceptibility of these two lines *in vitro* (Fig. 2B). Prior work has shown that isoforms with different PPQ resistance levels can transport this drug at similar levels in this assay format (16), suggesting a concentration-dependent mechanism of transport. Dd2 + F145I + C258W had reduced PPQ uptake, equal to half the level of Dd2 + F145I and consistent with the former isoform not conferring PPQ resistance. The 3D7 isoform demonstrated negligible transport at 100 nM ^3^H-PPQ, whereas the Dd2 isoform displayed low levels of transport at this PPQ concentration. This observation showed some contrast with the finding that parasites expressing either 3D7 or Dd2 PfCRT were PPQ-sensitive.

The CQ uptake data were more consistent with parasite susceptibility profiles (Fig. 2C and E; Table S3). The CQ-resistant Dd2 and the WT 3D7 isoforms exhibited the highest and lowest levels of CQ uptake, respectively. Dd2 + F145I PfCRT displayed reduced CQ transport, in agreement with the sensitization of Dd2^Dd2+F145I^ parasites to CQ. The Dd2 + F145I + F131C and Dd2 + F145I + I347T isoforms had a slight but insignificant increase in CQ uptake, concordant with parasite IC_90_ data. Dd2 + F145I + C258W PfCRT-containing liposomes accumulated the highest levels of CQ compared to Dd2 + F145I, although these were less than Dd2. As seen in other PfCRT transport studies, the 3D7 isoform had low levels of CQ transport, notwithstanding the sensitivity of WT parasites (31). These variations in PPQ and CQ transport likely reflect the differential involvement of these mutated amino acids in PfCRT-mediated drug transport. Of these four mutations, only F145I faces the inner cavity through which drug is transported (Fig. S4), which leads us to speculate that the other three mutations likely alter protein conformation to impact PPQ and/or CQ transport.

### Molecular dynamics energy minimization calculations provide evidence of conformational changes that would impact PfCRT interactions with drugs

**Fig 3:**
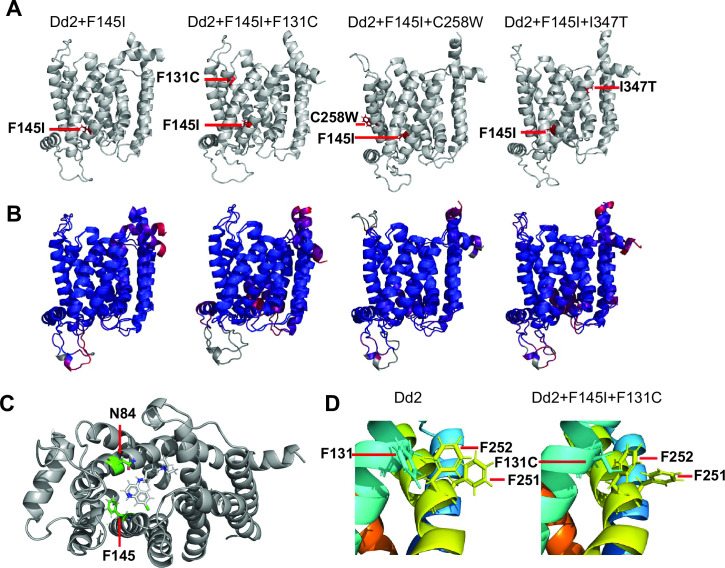
Molecular dynamics of the various PfCRT isoforms in this study. (**A**) Average energy minimized from cryoEM template (EMMD; see reference 32) structures of Dd2 + F145I, Dd2 + F145I + F131C, Dd2 + F145I + C258W, and Dd2 + F145I + I347T, with mutations highlighted relative to Dd2. (**B**) Root mean square deviation (RMSD) comparison of Dd2 PfCRT EMMD (32) vs Dd2 + F145I, Dd2 + F145I + F131C, Dd2 + F145I + C258W, or Dd2 + F145I + I347T EMMD structures (from left to right). Differences are highlighted using ColorByRMSD where blue indicates minimum RMSD and red indicates maximum RMSD. Gray regions were not compared. The structures are all highly similar with minimum RMSDs (0.18, 0.41, 0.11, and 0.39 Å from left to right), low average RMSDs (2.22, 2.44, 1.81, and 2.54 Å from left to right), and small regions of maximum RMSDs (11.91, 10.42, 9.44, and 9.73 Å from left to right) corresponding to particularly flexible PfCRT domains. (**C**) The previously proposed CQ binding site A in Dd2 showed direct interactions between the anilinyl CQ nitrogen (middle blue on the CQ molecule) and the oxygen of N84 (red) that is ~3.2 Å away. F145, positioned directly between the proposed CQ binding sites A and B, is likely to interact with the drug’s quinolinyl ring throughπ-π interactions during transit between the two sites. Notably, these π-π interactions were predicted to disappear upon mutation to isoleucine (F145I). The distance between the drug quinolinyl and F145 phenyl rings in site A is 7.4 Å. (**D**) The predicted three-phenylalanine π-π bundle for Dd2 PfCRT formed between the side chains of F131, F251, and F252 near the cytosolic ends of helices 3 (aqua) and 7 (yellow-green), respectively, is lost in the Dd2 + F131C + F145I isoform.

Subsequently, we used Monte Carlo Energy Minimization Molecular Dynamics to predict how the individual amino acid substitutions found in the three novel PfCRT isoforms might perturb local protein structure and drug interactions (32). As expected, the structures of the four PfCRT isoforms studied herein (Dd2 + F145I, Dd2 + F145I + F131C, Dd2 + F145I + C258W, and Dd2 + F145I + I347T) shared many similarities with the CQ-resistant Dd2 isoform from which they were derived (Fig. 3A and B). Nonetheless, the minor local differences between isoforms provide insight into their different PPQ binding or transport properties (PPQ is similar in structure to a CQ “dimer”), relative to CQ (18). To identify local differences in structure that may be relevant for the altered parasite phenotypes mediated by these novel PfCRT isoforms, we performed molecular dynamics analyses, as earlier applied to PfCRT to inventory residue side chain-side chain hydrogen bonds (HB) and salt bridges (SB) (32). Key differences between the isoforms are summarized in Table 2 with a full inventory of SB provided in Table S4.

**TABLE 2:** PfCRT isoform interactions^*a*^

	Interaction	Residue 1	Residue 2
All five isoforms	D57/K53	JM1	JM1
	E54/R392	JM1	TM10
	D311/K85	L7	L1
	E204/K200	L5	TM5
	D377/R374	TM10	L9
	E399/R392	TM10	TM10
Dd2 and Dd2 + F145I	D313/K317	L7	TM8
Dd2 only	D137/R231	TM3	TM6
	E232/K236	TM6	TM6
	D338/K339	TM8	TM8
	**Y68/D329**	TM1	TM8
	H97/S326*	TM2	TM8

^*a*^ List of interactions discussed in this study. HB are in boldface; SB are not. The interaction between H97 and S326 is present in all isoforms (*) but present for substantially less time for all F145I mutant isoforms relative to Dd2 PfCRT (≤30% vs 86% of simulation time).

Notably, all four isoforms lost key interactions within the “K76 region” that helps define drug binding site B, previously identified for the CQ-resistant Dd2 PfCRT (32). These include important Y68-D329 and H97-S326 HB present for Dd2 PfCRT but either absent or reduced to a minor contribution for all other isoforms, respectively (Table 2). In addition, three SB near the interface of the PfCRT three-helical “zipper” and the cytosolic end of the putative drug cavity are lost for all F145I mutant isoforms (Fig. S5). The F145I substitution in Dd2 PfCRT removed a phenyl aromatic ring (F145) that normally rests between drug binding sites A (at the entrance of the central cavity) and B (within this cavity about three helical turns away from site A). With F145, the ring is positioned directly within the cavity within ~7 Å of drug bound within site A (Fig. 3C) and is capable of π-π interactions with the CQ or PPQ quinoline ring during drug transit from site A to site B, whereas the alkyl side chain in 145I is not, likely influencing the energetics of the transition of CQ and PPQ drugs between the two binding sites.

Lastly, we observed an extended three-ring π-π stabilizing interaction for Dd2 PfCRT (Fig. 3D), which was conspicuously absent in the Dd2 + F145I + F131C isoform. Similar to the SB disruptions that appear to destabilize interactions between the cytosolic entrance to the PfCRT central cavity and the three-helix bundled PfCRT “zipper” noted earlier, the predicted loss of three-ring π-π interactions between residues 131, 251, and 252 likely perturbs binding of the zipper bundle to the cytosolic rim of the drug cavity. These simulations predict altered release of CQ and PPQ in these novel PfCRT isoforms relative to Dd2 PfCRT.

### Dd2 + F145I + C258W PfCRT results in decreased accumulation of short Hb-derived peptides in the DV

**Fig 4:**
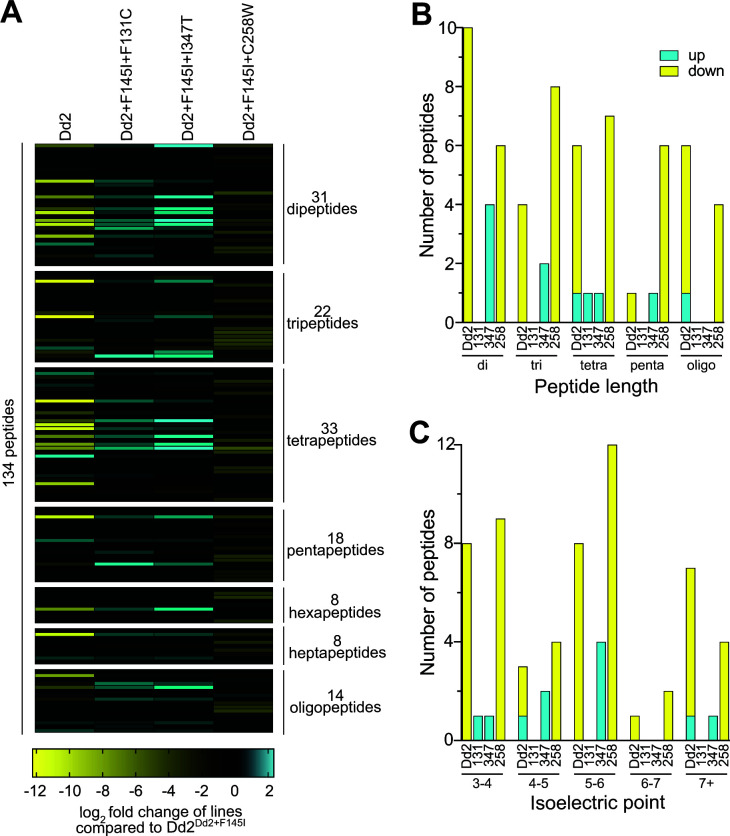
Dd2^Dd2+F145I+C258W^ results in decreased accumulation of peptides. (**A**) Heatmap of 134 peptides (detected in positive or negative modes and present in metabolite extracts from any of the parasite lines) classified by peptide length. These data are presented as the log_2_ fold change in the average abundance of each peptide identified compared to Dd2^Dd2+F145I^. *N* = 3; *n* = 3. Oligo refers to peptides eight or more amino acids in length. PfCRT haplotypes of the isogenic lines are listed. (**B and C**) Significant differences in peptide levels between lines with a given mutation and Dd2 + F145I are shown (*P* < 0.05 in unpaired *t*-tests). Peptides are classified by (**B**) peptide length or (**C**) isoelectric point. Details are provided in Tables S4 and S5.

Recent studies have linked PPQ resistance with excess accumulation of peptides resulting from aberrant Hb digestion and peptide processing in PfCRT mutants, including Dd2^Dd2+F145I^ (21). This is believed to modify DV physiology and affect access to amino acids for protein synthesis and intra-erythrocytic development. Given the increase in parasite fitness and alteration in DV size in our mutated F145I lines, we hypothesized that these novel PfCRT mutations may alleviate, to varying degrees, the buildup of peptides in this organelle. Peptidomic analysis, using liquid chromatography–mass spectrometry (LC-MS) revealed a total of 134 putative endogenous Hb-derived peptides, differing in both charge and length, identified in one or more samples (Table S5). We observed higher levels of peptides in Dd2^Dd2+F145I^ parasites compared to Dd2^Dd2^ (Fig. 4A), consistent with earlier findings (21). To determine the effect of the secondary mutations on peptide levels, each of the lines was compared to Dd2^Dd2+F145I^. Parasites harboring the F131C or I347T PfCRT mutations added to Dd2 + F145I accumulated slightly increased levels of short peptides (mostly two to five amino acids in length), exhibiting a similar phenotype to Dd2^Dd2+F145I^ (Fig. 4A). The Dd2^Dd2+F145I+C258W^ line, however, had markedly diminished levels of short peptides with over 75% of peptides decreased compared to Dd2^Dd2+F145I^ (Fig. 4A). We identified 27, 1, 8, and 31 differentially accumulated peptides in Dd2^Dd2^, Dd2^Dd2+F145I+F131C^, Dd2^Dd2+F145I+I347T^, and Dd2^Dd2+F145I+C258W^, respectively, which differed significantly from those in Dd2^Dd2+F145I^ (*P* ≥ 0.05 in unpaired *t*-tests; Fig. 4B and C; Table S6). Only five of the 27 peptides whose levels were altered in Dd2^Dd2^ were also increased in Dd2^Dd2+F145I+C258W^, suggesting that the C258W mutation alleviated peptide defects imparted by F145I, restoring regular peptide homeostasis (Fig. S6). Strikingly, all of the peptides with altered levels in Dd2^Dd2+F145I+C258W^ were reduced in abundance, which may explain the gain of fitness in this line relative to Dd2^Dd2+F145I^. Differentially accumulated peptides were typically two to four amino acids in length (Fig. 4B), had isoelectric points of three to six (Fig. 4C), and were neutral or carried a single negative charge at pH values of 5.5 or 7.4 (reflecting conditions in the DV lumen or cytosolic conditions, respectively) (Fig. S7), in accordance with prior studies (21, 33).

We used our proteoliposome system to explore whether peptides could compete for mutant PfCRT-mediated drug transport. These assays utilized the purified recombinant 7G8 + F145I and 7G8 isoforms, which mediate transport of ^3^H-PPQ and ^3^H-CQ, respectively (17). Assays focused on HVDDM and VDPVNF, as truncated or elongated versions of these peptides showed differences in Dd2^Dd2^ and Dd2^Dd2+F145I+C258W^, respectively, relative to Dd2^Dd2+F145I^ (Table S6). These two peptides were also previously found to inhibit ^3^H-CQ transport via Dd2 PfCRT expressed on the surface of *Xenopus* oocytes (34), and truncated versions were earlier reported to differ in their levels when comparing Dd2^Dd2^ with Dd2^Dd2+F145I^ (21). Both peptides showed substantial inhibition of ^3^H-PPQ or ^3^H-CQ transport, to levels similar to competitive inhibition with non-labeled PPQ or CQ (Fig. S8). Control assays showed no competition with pyrimethamine or atovaquone, unrelated drugs that are known to not interact with PfCRT.

## DISCUSSION

In this study, we characterized novel PfCRT mutations that emerged on the PPQ-R Dd2 + F145I PfCRT isoform—both with cultured parasites (F131C and I347T) and in SE Asian field isolates (C258W). Each of these mutations alleviated the fitness cost of Dd2 + F145I PfCRT to varying degrees and likely evolved to increase parasite replication rates in the absence of drug pressure. Both the C258W and I347T variants displayed reduced PPQ resistance, with the former being detected in SE Asia following the removal of PPQ as a first-line partner drug (27). Notably, the C258W field mutation restored CQ resistance to levels seen in Dd2 parasites, in contrast with the two mutations obtained during extended *in vitro* culture that conferred hypersensitization to CQ and md-CQ. Biochemical assays with purified PfCRT protein and peptidomic analysis revealed mechanistic differences that affect drug susceptibility, intracellular peptide availability, and parasite fitness.

Our data highlight the interplay between resistance, fitness, and drug usage that can impact which parasite genotypes predominate in individual regions. The introduction of DHA-PPQ in the late 2000s in Cambodia is known to have selected for novel PfCRT mutations, including F145I, in parasites expressing the CQ-resistant Dd2 isoform. These mutations have been shown to confer PPQ resistance *in vitro* and have been associated with an increased risk of treatment failure (9, 11, 12, 14, 35). Evidence suggests that F145I, which was among the earliest mutations and mediates high level resistance, was later replaced by the T93S, H97Y, and I218F mutations that, while less resistant, showed a smaller fitness cost (13, 15). These findings lead us to speculate that the emergence of the C258W mutation in the field and F131C and I347T in cultured parasites was a result primarily of improved fitness driving faster propagation rates (Fig. 1). As has been seen with CQ resistance, even a marginal increase in growth rate per generation can allow a parasite strain to gain prevalence. Earlier studies in Malawi have shown that the fitness costs observed with CQ-resistant PfCRT isoforms was sufficient for WT, CQ-sensitive parasites to quickly overtake mutant lines in the absence of CQ usage in clinics (36, 37). Recent data from the MalariaGEN Pf7 genome study provide evidence of a similar trajectory with mutant PfCRT in SE Asia, as the parasite evolves away from PPQ-R PfCRT isoforms that impart a substantial fitness cost (27).

The identification of distinct mutations in the field and in lab-cultured parasites suggests multiple evolutionary paths for PfCRT. We observed a range of PPQ phenotypes with our original clones and edited lines that inversely correlated with growth rate, i.e., the more resistant parasites displayed a stronger fitness cost (Fig. 1B and Fig. 2A). The F131C mutants maintained high survival rates in the PSA with only a small improvement in growth rate per generation compared to Dd2^Dd2+F145I^ (~2%), whereas the I347T parasite lines became fully sensitized to PPQ but had up to a twofold increase in replication rate. The C258W variant had a more moderate increase in fitness despite a complete loss of PPQ resistance, as evidenced by decreased PSA and PPQ IC_90_ values. These data illustrate the often dichotomous relationship between parasite fitness and increased PSA survival rates (38). Observations from these edited lines may provide lessons on the evolution of the SE Asian parasite landscape after DHA-PPQ cessation, pointing to a rapid loss of resistance. These data also underscore the necessity for routine surveillance of the entire *pfcrt* locus. Importantly, the presence of the F145I point mutation does not guarantee PPQ resistance.

Strikingly, Dd2 + F145I + C258W PfCRT mediates CQ resistance, despite the presence of the F145I mutation and lack of CQ pressure in SE Asia (Fig. 2C). PPQ-R mutations, which line the drug-transporting central cavity of PfCRT, generally hypersensitize the parasite to CQ (12). The C258W mutation faces away from this cavity (Fig. S4), potentially causing a conformational change that affects both PPQ and CQ transport. The elevated CQ IC_90_ of this field line is important to note given the recent modeling data suggesting that simultaneous administration of PPQ and CQ could preclude the acquisition of dual drug resistance (16). Critically, there was no change in parasite susceptibility to MFQ, the first-line ACT partner drug in Cambodia, in any of the mutant lines (Fig. S3F).

Our transport studies with purified PfCRT variants reveal notable differences between CQ and PPQ, despite these both being 4-aminoquinoline compounds (PPQ is a bis-4-aminoquinoline with two CQ-like structures connected by a central linker). For CQ, transport levels were consistent with the CQ inhibitory values against resistant and sensitive parasite lines, highlighting drug efflux as the primary determinant of resistance (Fig. 2C and E) (39). For PPQ, however, transport and susceptibility data do not match as closely. At 100 nM ^3^H-PPQ, the Dd2 + F145I, Dd2 + F145I + F131C, and Dd2 + F145I + I347T isoforms transported comparable amounts of drug, despite the PPQ-sensitive phenotype of the I347T variant (Fig. 2B and D). This difference has also been observed with ^3^H-CQ and ^3^H-PPQ cellular accumulation studies in the parasite (17). One possible explanation for the differences in CQ and PPQ transport could be due to the increased protonation state of PPQ. Inside the DV (whose pH is estimated to be ~5.2 40]), CQ would be protonated at both its nitrogens, whereas PPQ can have up to four protonated nitrogens. CQ transport via mutant PfCRT was earlier shown to be dependent on H^+^ co-transport (41). We speculate that, once PPQ reaches a certain concentration in the DV, there may be sufficient protons to permit efficient transport without requiring external protons. In the parasite, PPQ resistance is evident only at high concentrations of drug, manifesting at the IC_90_ but not IC_50_ level. One caveat of our study is that transport measurements were conducted at a single concentration (100 nM ^3^H-PPQ). A recent study observed growth inhibition of PfCRT-expressing *Saccharomyces cerevisiae* yeast only at levels of 300 µM PPQ as a surrogate of transport measurement (18). Technical considerations preclude using high concentrations of ^3^H-PPQ in our assays.

Molecular dynamics simulations uncovered subtle differences between isoforms that were predicted to result in the loss of several key molecular interactions in the central cavity of PfCRT (Fig. 3). This includes the loss of SB involving residues on TM3, TM6, and TM8 near the cytosolic entrance of the central drug-binding cavity, likely providing greater helical flexibility to facilitate release of the larger PPQ molecule as opposed to the smaller CQ (32). Also, another perturbation involves residue 131 (i.e., the mutated amino acid in the PPQ-R Dd2 + F145I + F131C). Previous molecular dynamics studies have suggested that the F145I mutation induces a conformational change in PfCRT, attributing PPQ resistance to these structural alterations as opposed to changes in electrostatic properties (16, 17). Our study now predicts the disappearance of π-π interactions with PPQ upon the replacement of the aromatic phenylalanine with isoleucine. These molecular dynamics simulations support the hypothesis that CQ and PPQ binding within PfCRT involves different side chain interactions and conformations of the protein (19) and predict that a given point mutation can lead to small changes in structure that result in larger changes in drug susceptibility phenotypes.

In previous metabolomic analyses, Dd2^Dd2+F145I^ accumulated high levels of Hb-derived peptides (21), a phenotype that was replicated in our study. Given this, we hypothesized that the physiological rationale for acquiring second-site mutations may be to alleviate this buildup of peptides. This was true for the Dd2^Dd2+F145I+C258W^ line, with the majority of Hb-derived peptides showing relatively reduced abundance (Fig. 4A). Among the 31 peptides that showed a significant decrease compared to Dd2^Dd2+F145I^ (Fig. 4B and C; Table S6), we observed PVNF and HVDD, as well as the dipeptides VG, GV, NP, and PA, which have been identified in earlier PfCRT studies (21, 34). PfCRT is thought to transport Hb-derived peptides from the DV lumen to the cytosol (34). It is possible that the C258W mutation emerged in field isolates to correct impaired peptide efflux by Dd2 + F145I PfCRT, thereby increasing amino acid availability in the cytosol and improving parasite replication rates (42). We note that most of the previously identified peptides were short (4–11 amino acids in length) and were neutral or had a single positive charge, in accordance with the increased pool of peptides in the Dd2^Dd2+F145I+C258W^ line. In addition, this excess of peptides in the DV likely causes bloating of this organelle, as exhibited by Dd2^Dd2+F145I^ but not Dd2^Dd2+F145I+C258W^ (Fig. S2), which we suspect might lead to increased osmotic pressure and decreased proliferative potential.

Interestingly, quantitative peptidomics did not reveal a major difference in peptide accumulation in Dd2^Dd2+F145I+F131C^ and Dd2^Dd2+F145I+I347T^ relative to Dd2^Dd2+F145I^, with only one or eight peptides significantly altered in these lines, respectively (Fig. 4B and C; Table S6). In both lines, schizonts still had bloated vacuoles, further confirming a buildup of peptides in the DV (Fig. S2). A reduced accumulation of short peptides, however, may not fully explain the gain in fitness in these parasites. PfCRT mutations have also been associated with defective Hb catabolism and alterations in ionic balance (21, 43), which may impact fitness. Further studies on these lines will be necessary to determine additional physiological effects of these mutations and how they alter ABS parasite fitness.

Our data underscore how changes in parasite fitness and antimalarial susceptibility can drive parasite genetic evolution in endemic areas with differing drug selective pressures. PPQ-R parasites appear to be highly transient in the absence of sustained PPQ pressure, due to the large fitness cost of their mutations. Reduced fitness has also been implicated with multicopy *plasmepsin II/III* that co-evolved with PPQ-R PfCRT isoforms in the field and that appear to augment resistance (13, 26). In light of these data, DHA-PPQ may be a good candidate as part of a multiple first-line therapy strategy (i.e., used simultaneously with a separate ACT) in SE Asia to delay the emergence of resistance (44–47). Nonetheless, as different drug regimens are implemented in SE Asia, it will be imperative to survey the entire *pfcrt* allele to ascertain resistance status, as the presence of a single point mutation does not guarantee resistance to PPQ or sensitivity to CQ.

## MATERIALS AND METHODS

### Whole-genome sequencing

Sequencing of genomic DNA from mutant and parental lines employed an Illumina TruSeq DNA PCR-Free library preparation protocol and a MiSeq sequencing platform, as described (48). The list of variants was compared against the Dd2 +F145I parent to obtain homozygous SNPs that were present exclusively in the clones.

### Plasmid construction, parasite culturing, and transfections

*pfcrt* was edited using customized zinc finger nucleases and a two-plasmid approach that replaces the endogenous allele with a recombinant allele containing the mutations of interest (28). Mutations were introduced into a *pfcrt*^Dd2^ donor plasmid by site-directed mutagenesis (Fig. S1 and Table S7). Dd2 *P. falciparum* ABS parasites were cultured in human O^+^ red blood cells (RBCs) in RPMI 1640-based culture media containing 0.5% (wt/vol) Albumax (49). RBCs were purchased from the Interstate Blood Bank (Memphis, TN) as pooled, de-identified, anonymized blood that was washed to remove any residual leukocytes. Approval for this protocol (AAAU3761) was provided on 28 October 2022 by the Columbia University Institutional Review Board, which deemed this work to be Not Human Subjects Research Under 45 CFR 46.

Parasites were cultured at 37°C in an airtight chamber with 5% O_2_/5% CO_2_/90% N_2_. Ring-stage parasites were electroporated with 50–100 µg of purified plasmid DNA in Cytomix (49). The donor plasmid carrying the human *dhfr* marker was selected with 2.5 nM WR99210 (Jacobus Pharmaceuticals), and the zinc finger nuclease plasmid expressing blasticidin S-deaminase was selected with a 6-day pulse of 2 µg/mL blasticidin hydrochloride (Thermo Fisher). Editing was confirmed using PCR primers p1–7 (Fig. S1; Table S7) and Sanger sequencing, and clones were obtained by limiting dilution.

### *In vitro* fitness assays

We measured relative growth rates of *pfcrt*-edited parasite lines using *in vitro* competition assays (10, 12). Briefly, parasites were co-cultured in 96-well plates in a 1:1 ratio with a Dd2 reporter parasite line (termed Dd2-GFP) that expresses eGFP (24). Parasites were stained with MitoTracker Deep Red and growth monitored for 21 days on an iQue flow cytometer. Relative growth rates of each test line are shown by plotting the percentage of GFP^+^ cells in culture over time. The fitness cost associated with a line expressing a given PfCRT mutation was calculated relative to the Dd2 GFP^+^ reporter line using the following formula: *P*′ = *P*[(1 − *x*)*^n^*], where *P*′ is equal to the parasitemia at the assay endpoint, *P* is equal to the parasitemia on day 0, *n* is equal to the number of generations from the assay start to finish, and *x* is equal to the fitness cost (50). This equation assumes 100% growth for the GFP^+^ comparator line. The assay endpoint was defined as the final day of the experiment day 21, resulting in the number of parasite generations (*n*) being set to 10.5. From these data, we determined the fitness cost relative to Dd2^Dd2^.

### Piperaquine survival assay

For PSAs, we seeded tightly sorbitol-synchronized ring-stage parasites (0–6 h post invasion) at 1% parasitemia and 1% hematocrit in 96-well flat-bottom plates containing 10-point, twofold dilutions with a maximum of 1,600 nM PPQ (12, 13, 30). Parasites were incubated for 72 h at 37°C. Parasites were labeled with SYBR Green I and MitoTracker Deep Red (as DNA and mitochondrial dyes, respectively), and parasitemias were measured on an iQue Plus flow cytometer. Percent survival was calculated by dividing the parasitemia of the PPQ-treated parasites by that of the no-drug control (10, 12). Statistical significance was determined using non-parametric Mann-Whitney *U* tests (GraphPad Prism 8 software). Raw data and statistics are listed in Table S1.

### Drug susceptibility assays

Asynchronous, ABS parasites were plated at 0.3%–0.5% parasitemia and 1% hematocrit in 96-well plates and incubated with a 10-point, twofold range of drug concentrations. Plates were incubated at 37°C for 72 h, and parasitemias were measured by flow cytometry. IC_50_ values were calculated by nonlinear regression analysis. Statistical significance was determined using Mann-Whitney *U* tests (Table S2).

### PfCRT protein expression and purification

The *pfcrt* full-length open-reading frames were cloned into the pEG BacMam vector (51), and recombinant P3 baculovirus was prepared as described in reference 17. Proteins were expressed in HEK293S GnTi-negative cells and purified by Ni^2+^-NTA resin chromatography (17, 52).

### Transport assays

Purified PfCRT variants were reconstituted in preformed liposomes (composed of *E. coli* total lipids:CHS at a ratio of 94:6 [wt/wt] and a protein:lipid ratio of 1:150 [wt/wt]). Each uptake assay was performed with 50 ng of PfCRT in proteoliposomes, and 1-min uptakes of ^3^H-CQ (50–100 nM, 1 Ci/mmol) or ^3^H-PPQ (50–100 nM, 1 Ci/mmol; both from American Radiolabeled Chemicals, Inc.) were performed as described (17). Statistical significance was determined using Mann-Whitney *U* tests (Table S3).

### Simulations of molecular dynamics

Monte Carlo Molecular Dynamics (MC/MD) energy minimization computations for the PfCRT Dd2 isoform have been published previously (32). MC/MD energy minimization for the four additional PfCRT protein isoforms analyzed here (Dd2 + F145I, Dd2 + F145I + F131C, Dd2 + F145I + C258W, and Dd2 + F145I + I347T) was performed essentially as described previously for Dd2. In brief, we first imported the structure of the PfCRT Dd2 isoform solved by mutagenesis and MC/MD methods, based on the 7G8 PfCRT isoform cryo-EM structure template (PDB code: 6UKJ), into Maestro (53). The additional PfCRT isoforms (Dd2 + F145I, Dd2 + F145I + F131C, Dd2 + F145I + C258W, and Dd2 + F145I + I347T) were generated using Maestro’s Residue and Loop Mutation tool. Each isoform was then prepared using Protein Preparation Wizard to bond orders. We added missing hydrogen atoms using Prime Protonation states of ionizable residues, fixed at pH 5.0 (near the pH of the DV), using ProtAssign. Each isoform was embedded within a 1-palmitoyl-2-oleoyl-sn-glycero-3-phosphocholine (POPC) membrane (54). Boundary conditions were an orthorhombic box expanding 10 Å beyond the protein in the X, Y, and Z dimensions (membrane defines the X,Y plane), solvated with simple point-charge water, and neutralized with chloride ions. MC/MD simulations each lasted 10 ns (55). Three independent simulations were done for each isoform and then averaged before analysis and comparison to Dd2 PfCRT. Each calculation began with default relaxation followed by simulation in an isothermal, isobaric NPT ensemble with constant particle number (N), pressure (P; 1.01325 bar), and temperature (T; 310 K) (32). Convergence for each simulation was typically observed within 2–4 ns.

To analyze isoforms, clusters were created for each MC/MD trial and combined, and all atoms then included as the root mean square deviation (RMSD) matrix. Clustering was with the Desmond Trajectory Clustering tool (56), and water and POPC membrane were manually removed with Maestro for ease of visual comparison. To compare PfCRT isoforms, all-atom RMSD was generated, and local structural differences were visualized using PyMOL ColorByRMSD (57). SB and side chain-side chain HB differences were identified using Visual Molecular Dynamics (VMD) software (58) as described (32). After scanning the entire protein, HB were defined as hetero-atom distances within 3.2 Å with bond angle 180° ± 45°, and SB, as hetero-atom interactions used a cutoff of 4 Å. Heatmaps summarizing pair interaction were generated using VMD.

### Sample preparation for untargeted LC-MS metabolomics

*Mycoplasma*-free parasites were synchronized to trophozoites using sorbitol, followed by MACS CS columns on a SuperMACS II Separator (Miltenyi Biotec) to remove uninfected RBCs. Hydrophilic parasite metabolites were extracted using 1-mL 90% cold methanol with 0.5 µM [^13^C_4_, ^15^N_1_]-aspartate (Cambridge Isotope Laboratories) as the internal standard to correct for technical variations arising from sample processing. Cell pellets were disrupted by thoroughly vortexing the sample tubes, the insoluble debris was pelleted by centrifugation (13,000 × *g*, 10 min), and the supernatant was collected and dried down under nitrogen gas flow. Samples were then resuspended in HPLC-grade water containing 1 µM chlorpropamide (Alpha Aesar) as an internal standard to correct for signal deviation due to instrument variance. A quality control sample consisting of all samples pooled together was created and periodically measured throughout the run to monitor signal consistency throughout the otherwise randomized run order.

### LC-MS parameters and methods

Five microliters of each sample were injected for analysis. Metabolites were analyzed using a reversed phase method on an HPLC Prominence 20 UFLCXR system (Shimadzu) with a Waters BEH C18 column (100 mm × 2.1 mm, 1.7-µm particle size) at 55°C and an aqueous acetonitrile gradient run for 20 min at a flow rate of 250 µL/min. Solvent A was an HPLC-grade water with 0.1% formic acid, and solvent B was an HPLC-grade acetonitrile with 0.1% formic acid. The solvent gradient was as follows: at 0.0 min, 3% of B; 10.0 min, 45% of B; 12.0 min, 75% of B; 17.5 min, 75% of B; and 18.0–20.0 min, 3% of B. Eluate was delivered into a (QTOF) 5600 TripleTOF using a DuoSpray ion source (AB Sciex). Capillary voltage was 5.5 kV in positive and 3.8 kV in negative ion mode with declustering potentials of 80 V and −80 V, respectively. During the analysis, curtain gas pressure of 25 psi, nebulizer gas (GS1) of 50 psi, heater gas 2 (GS2) of 50, and heater temperature of 600°C were applied. The TripleTOF was scanning 50 to 1,000 m/z, and 16 MS/MS product ion scans (100 ms) per duty cycle using collision energy of 50 V with a 20-V spread.

### Data analyses for metabolomics

Analyses were performed as previously described (21). The labeled [^13^C_4_, ^15^N_1_]-aspartate internal standard intensity was assessed for technical reproducibility. Peak areas from both positive and negative modes were exported, and the resulting feature quantification matrices were used as input for a custom R script designed to putatively annotate peak groups as human Hb-derived peptides of matching m/z value within 15 ppm, considering all potential human Hb peptides up to 13 amino acids long. Peak areas from both positive and negative modes were exported for downstream analysis. The chlorpropamide standard was used as an internal control to normalize individual metabolite peak areas between runs. The peak areas for duplicate metabolites were summed before blank subtraction. Peak areas of the blanks were subtracted from the samples for each metabolite and in each experiment. The relative standard deviation of quality controls was tested, and only peptides with a value <30 were retained. The isoelectric point and charge at pH 5.5 and pH 7.4 for each oligopeptide were calculated using the web-based isoelectric point calculator IPC2.0 (59). Data from three independent experiments, each with three technical replicates, were averaged. To investigate the effect of PPQ-R PfCRT mutations on *P. falciparum* metabolism, the log_2_ fold changes of mutant vs Dd2^Dd2+F145I^ metabolites were calculated for each experimental run using the averaged replicate peak areas. Raw data are listed in Table S5. The Venn diagram in Fig. S6 was made using the website http://bioinformatics.psb.ugent.be/webtools/Venn/.

## SUPPLEMENTAL MATERIAL

Supplemental Material mbio.01832-23-s0001.pdf Fig S1-S8 and Tables S1-S7.

ASM does not own the copyrights to Supplemental Material that may be linked to, or accessed through, an article. The authors have granted ASM a non-exclusive, world-wide license to publish the Supplemental Material files. Please contact the corresponding author directly for reuse.
