# Supplementary material for: Additional PfCRT mutations driven by selective pressure for improved fitness can result in the loss of piperaquine resistance and altered Plasmodium falciparum physiology
Source: mBio. 2023 Dec 7;15(1):e01832-23. doi: 10.1128/mbio.01832-23 (PMC10790694; doi:10.1128/mbio.01832-23)
Supplement: Supplemental Material — Fig S1-S8 and Tables S1-S7. [file mbio.01832-23-s0001.pdf]

Supplemental Material

Additional PfCRT Mutations Driven By Selective Pressure for Improved Fitness Can Result in the Loss of Piperaquine Resistance and Altered *Plasmodium falciparum* Physiology

Laura M. Hagenah, Satish K. Dhingra, Jennifer L. Small-Saunders, Tarrick Qahash, Andreas Willems, Kyra A. Schindler, Gabriel W. Rangel, Eva Gil-Iturbe, Jonathan Kim, Emiliya Akhundova, Tomas Yeo, John Okombo, Filippo Mancia, Matthias Quick, Paul D. Roepe, Manuel Llinás, David A. Fidock

Supplemental Figures and Tables

Figure S1: Zinc-finger nuclease (ZFN)-mediated editing of *pfcrt*……………………………………………….page 2
Figure S2: Cell morphology of *pfcrt*-edited parasites ……...page 3
Figure S3: IC_{50} values for relevant antimalarials………………………………..................................page 4
Figure S4: Mapping of mutations onto the PfCRT structure.........……………………………………….page 5
Figure S5: Visual representation of salt bridge interactions broken as Dd2 PfCRT evolves to any of the F145I mutant isoforms……………………………………………………………………………………...page 6
Figure S6: Venn diagram of numbers of significantly altered peptides ……….......................................page 7
Figure S7: Number of differentially accumulated peptides shown as a function of peptide charge of accumulated peptides at pH 5.5 or 7.4 ………...page 8
Figure S8: Peptides HVDDM and VDPVNF inhibit ^{3}H-PPQ and ^{3}H-CQ transport via PfCRT……………page 9
Table S1: Piperaquine survival assay (PSA) values of *pfcrt*-modified parasite lines………………………page 10
Table S2: Mean IC_{50} and IC_{90} values of *pfcrt*-modified parasite lines…………………….........................page 11
Table S3: Transport in proteoliposomes……………..……...……………………………..........................page 12
Table S4: All salt bridges found for all PfCRT isoforms………...……...……...…….………………………page 13
Table S5: Averaged log_2 fold change of the baseline peptide levels in the variant PfCRT lines versus Dd2$^{Dd2+F145I}$……………………..page 14
Table S6: List of peptides showing significantly different levels in Dd2^{Dd2}, Dd2$^{Dd2+F145I+F131C}$, Dd2$^{Dd2+F145I+I347T}$, and Dd2$^{Dd2+F145I+C258W}$ compared to Dd2$^{Dd2+F145I}$……………………………………………..page 16
Table S7: List of oligonucleotides used in this study…………………………………………………………page 18

SUPPLEMENTAL REFERENCES……………………………………………………………………………page 19

Figure S1

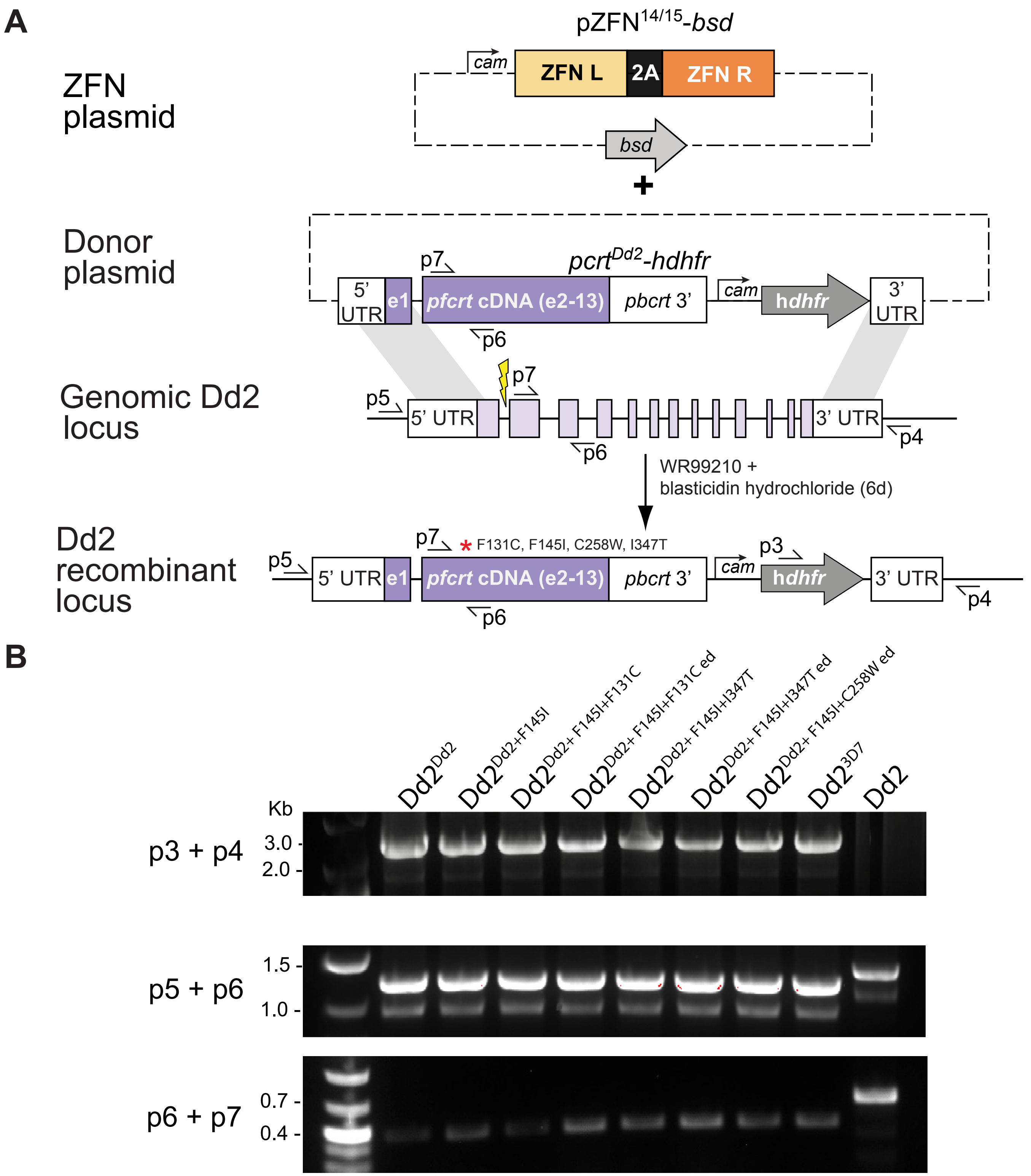

Fig. S1. Zinc-finger nuclease (ZFN)-mediated editing of *pfcrt*. (**A**) The *pfcrt* gene was edited using a two-plasmid approach, one containing the homologous donor template and the other containing the *pfcrt*-specific pair of ZFNs linked by a 2A ribosome skip peptide (1). Parasites were selected for the human dihydrofolate reductase (h*dhfr*) and the blasticidin S-deaminase (BSD) markers with WR99210 and blasticidin hydrochloride, respectively. (**B**) Three sets of PCRs were performed to confirm editing and the modified locus was verified by Sanger sequencing. Primer locations are denoted in (**A**), and sequences are listed in **Table S7**.

Figure S2

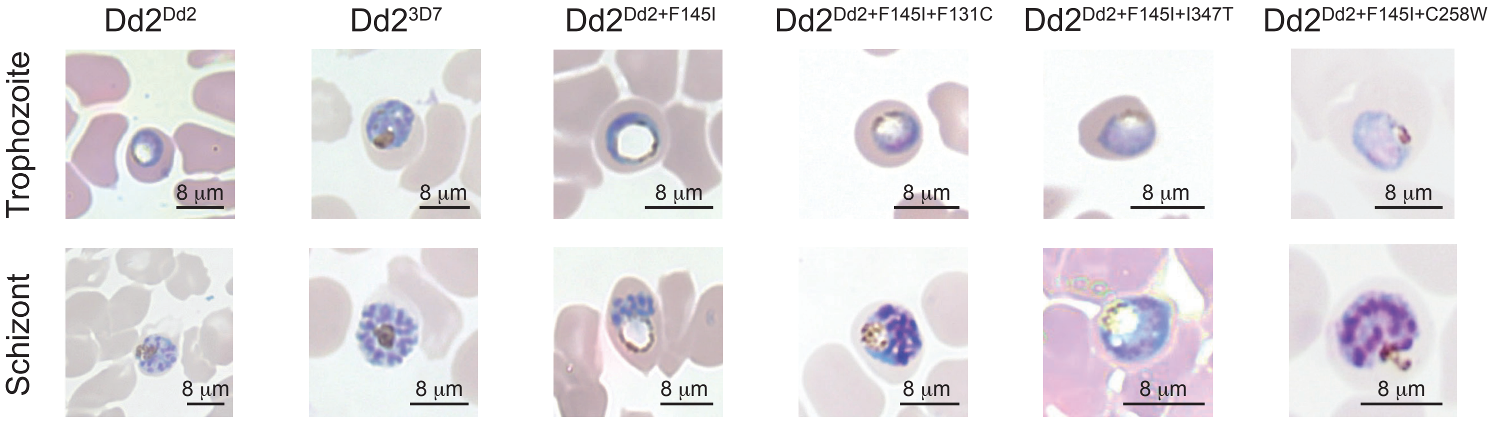

Fig. S2. Cell morphology of *pfcrt*-edited parasites. $Dd2^{Dd2+F145I}$ has distended digestive vacuoles characteristic of piperaquine-resistant parasites in both the trophozoite and schizont asexual blood stages. $Dd2^{Dd2+F145I+F131C}$, $Dd2^{Dd2+F145I+I347T}$, and $Dd2^{Dd2+F145I+C258W}$ vacuoles appear smaller compared to $Dd2^{Dd2+F145I}$. Note that the partially bloated vacuoles evident in $Dd2^{Dd2+F145I+F131C}$ and $Dd2^{Dd2+F145I+I347T}$ associate with a minimal change in peptide accumulation relative to $Dd2^{Dd2+F145I}$ (**Fig. 4**).

Figure S3

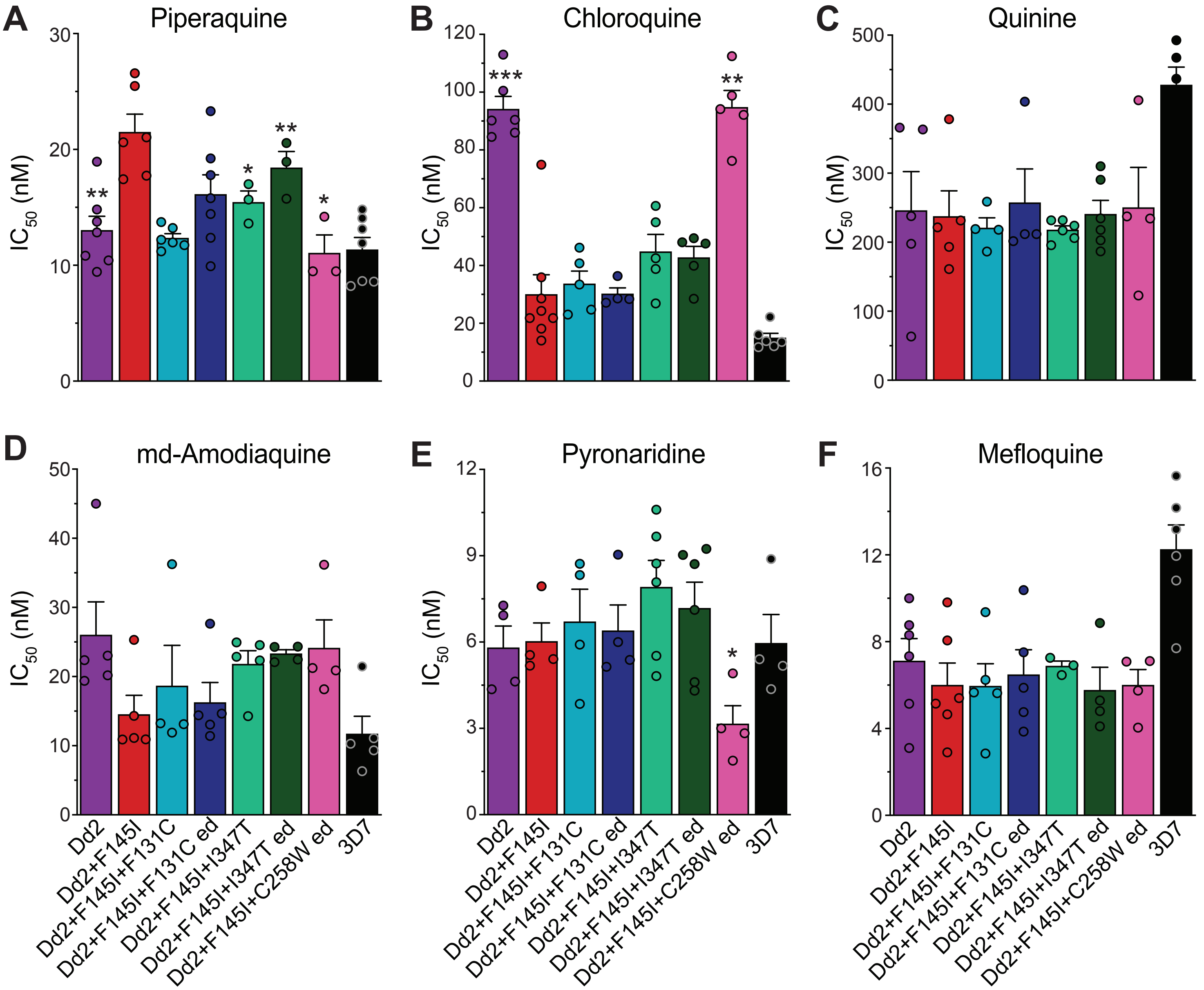

Fig. S3. IC_{50} values for relevant antimalarials. Mean ± SEM IC_{50} values (**Table S2**) were calculated from 72-hr dose-response assays for: (**A**) Piperaquine; (**B**) Chloroquine; (**C**) Quinine; (**D**) monodesethyl (md)-Amodiaquine; (**E**) Pyronaridine; and (**F**) Mefloquine. N, n = 4-7, 2. Statistical significance was determined using Mann-Whitney *U* tests as compared to the isogenic $Dd2^{Dd2+F145I}$ line. **P* < 0.05; ***P* < 0.01; ****P* < 0.001. Individual circles indicate values from each independent experiment.

Figure S4

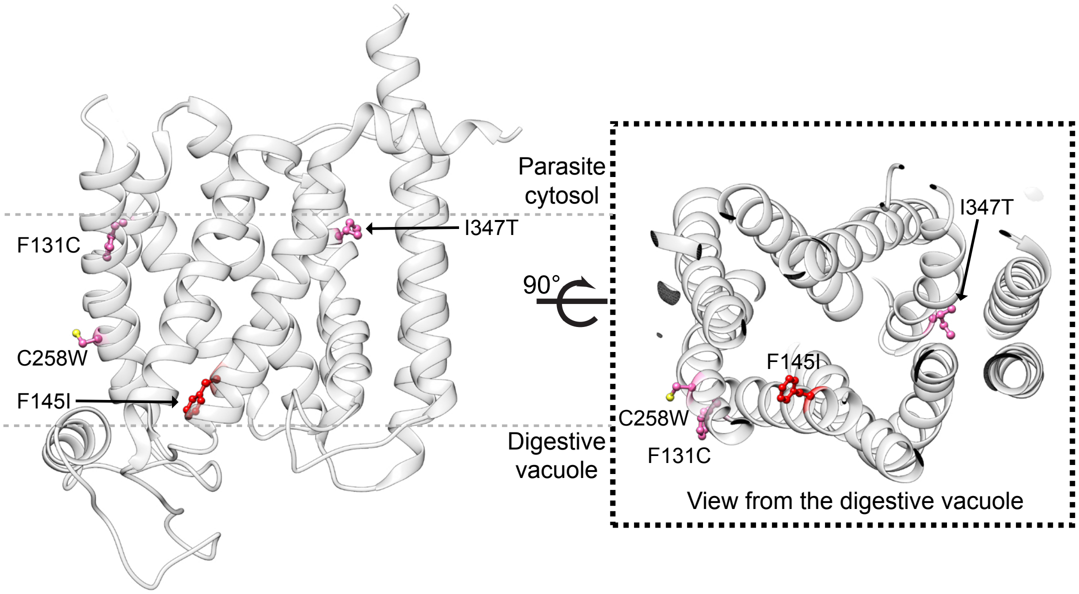

Fig. S4. Mapping of mutations onto the PfCRT structure. F145I, F131C, C258W, and I347T are mapped onto the known 7G8 cryo-EM structure (2). Mutations have their side chains rendered as sticks and are colored in red (F145I) or pink (F131C, C258W and I347T). The remaining structures are rendered in cartoon and colored in white. Views are shown vertically (digestive vacuole to the bottom) and rotated to show the structure from the digestive vacuole side.

Figure S5

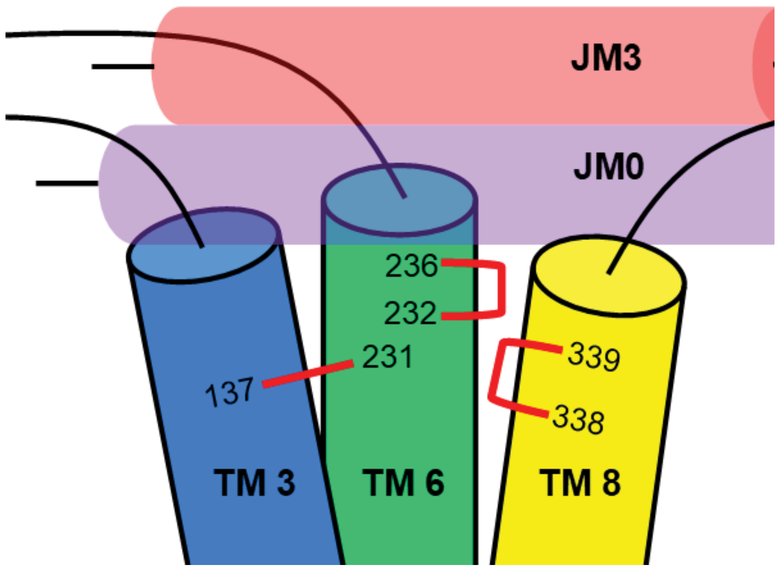

Fig. S5. Visual representation of salt bridge interactions broken as Dd2 PfCRT evolves to any of the F145I mutant isoforms. Transmembrane (TM) helix numbers are denoted, with PfCRT "zipper" bundled helices JM3 and JM0 shown above [3]. The full list off salt bridge interactions is listed in **Table S4**.

Figure S6

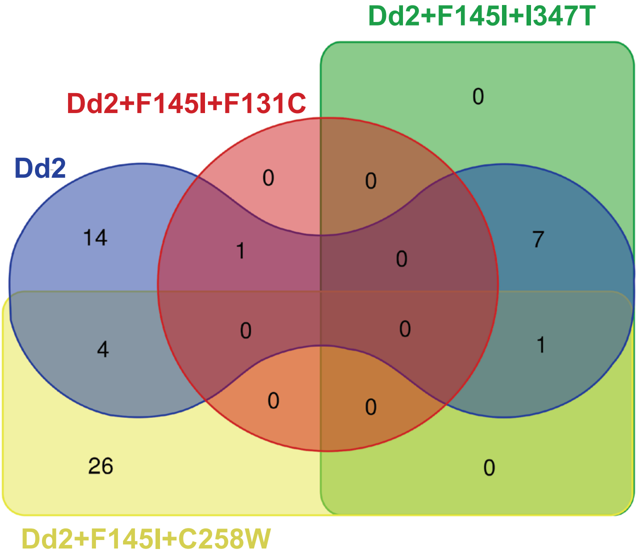

Fig. S6. Venn diagram of numbers of significantly altered peptides. Venn diagram showing numbers of peptides whose levels were significantly increased or decreased peptides for the lines $Dd2^{Dd2}$ (blue), $Dd2^{Dd2+F145I+F131C}$ (red), $Dd2^{Dd2+F145I+I347T}$ (green), or $Dd2^{Dd2+F145I+C258W}$ (yellow), relative to the isogenic $Dd2^{Dd2+F145I}$ line. The largest numbers of differences were observed with $Dd2^{Dd2+F145I+C258W}$ and $Dd2^{Dd2}$, as reflected in the heatmap (**Fig. 4A**) that showed these two lines being the most divergent compared with $Dd2^{Dd2+F145I}$. Significance was attributed to a peptide when it showed a statistically significant difference ($P < 0.05$) between the $Dd2^{Dd2+F145I}$ reference line and an isogenic test line (Student's *t* test, data obtained from 3 independent experiments; see **Table S6**).

Figure S7

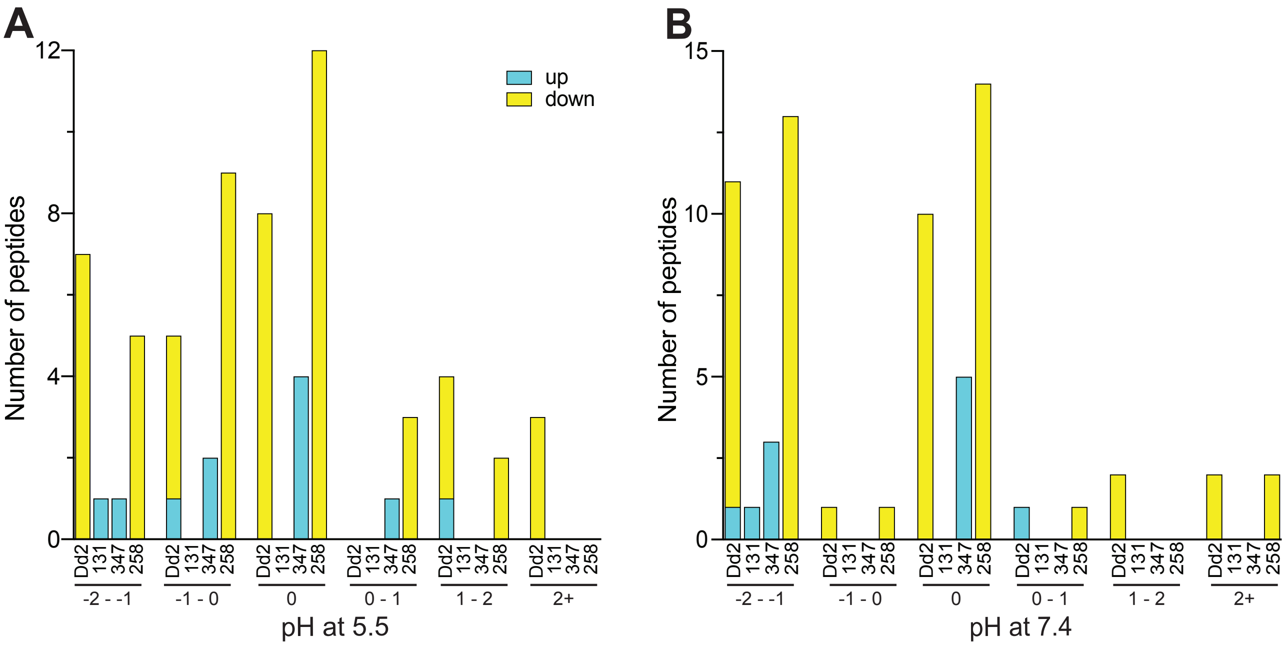

Fig. S7. Number of differentially accumulated peptides shown as a function of peptide charge of accumulated peptides at pH 5.5 or 7.4. Plots show differences in peptide levels between lines with a given mutation and Dd2$^{Dd2+F145I}$. Peptides are classified by (**A**) charge at pH 5.5 (representing the DV lumen) or (**B**) charge at pH 7.4 (representing the cytosol). Details are provided in **Table S6**.

Figure S8

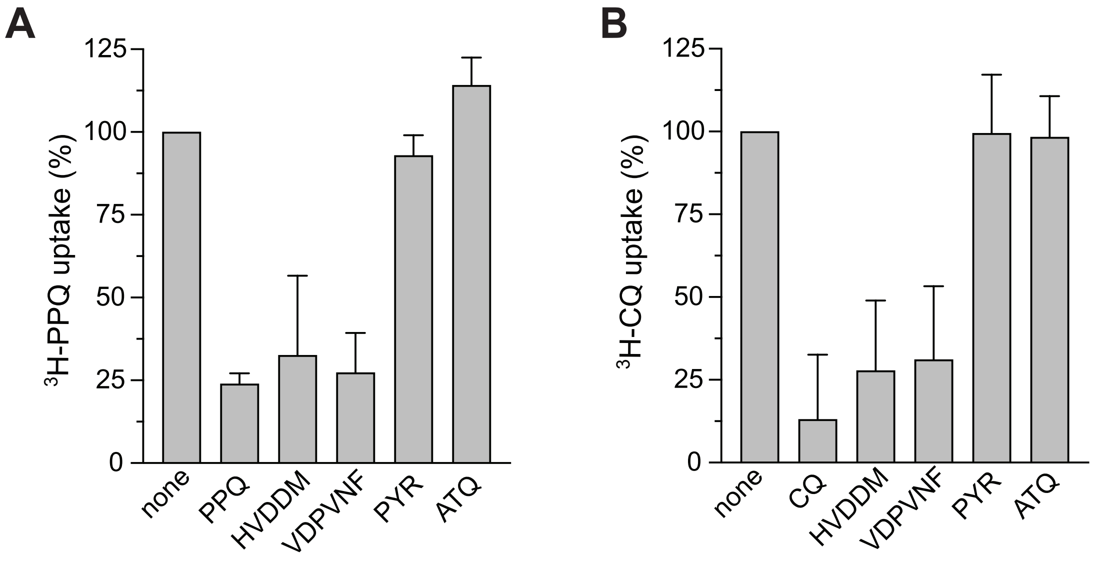

Fig. S8. Peptides HVDDM and VDPVNF inhibit ^{3}H-PPQ and ^{3}H-CQ transport via PfCRT. Uptake of (**A**) 50 nM ^{3}H-PPQ by 7G8+F145I PfCRT or (**B**) ^{3}H-CQ by 7G8 PfCRT was measured for 1 min in the presence or absence of the 25 µM HVDDM or VDPVNF. Values were normalized to the signal in the absence of the non-radiolabelled compound (”none”). CQ, PPQ, and the non-PfCRT related drugs pyrimethamine (PYR) and atovaquone (ATQ) were also tested at 25 μM. Data are means ± s.d. of 3 independent experiments.

Table S1 Piperaquine survival assay values of *pfcrt*-modified parasite lines.

	**Dd2^{Dd2}**	**Dd2^{3D7}**	**Dd2$^{Dd2+F145I}$**	**Dd2$^{Dd2+F145I+F131C}$**	**Dd2$^{Dd2+F145I+F131C}$ (edited)**	**Dd2$^{Dd2+F145I+I347T}$**	**Dd2$^{Dd2+F145I+I347T}$ (edited)**	**Dd2$^{Dd2+F145I+C258W}$ (edited)**
**1600 nM**	**3.5 ± 0.7**	**2.9 ± 1.5**	**35.0 ± 2.0**	**28.4 ± 3.6**	**33.6 ± 1.0**	**6.7 ± 1.1**	**13.3 ± 1.9**	**3.2 ± 0.6**
N	4	6	7	7	8	3	2	3
P vs Dd2^{Dd2}	–	0.61	0.006	0.006	0.010	0.11	0.13	>0.99
P vs Dd2$^{Dd2+F145I}$	0.006	–	–	0.24	0.51	0.056	0.017	0.017
**800 nM**	**3.1 ± 1.1**	**3.3 ± 0.5**	**32.9 ± 3.0**	**28.5 ± 2.9**	**30.5 ± 2.0**	**2.9 ± 1.5**	**5.9 ± 4.5**	**3.3 ± 0.7**
N	4	6	7	7	7	3	2	3
P vs Dd2^{Dd2}	–	>0.99	0.006	0.006	0.006	0.86	0.80	0.86
P vs Dd2$^{Dd2+F145I}$	0.006	–	–	0.30	0.59	0.017	0.056	0.017
**400 nM**	**3.3 ± 0.8**	**2.5 ± 0.5**	**23.1 ± 2.4**	**18.8 ± 3.2**	**19.2 ± 2.3**	**1.9 ± 0.3**	**2.9 ± 0.6**	**3.3 ± 0.7**
N	4	6	7	7	8	3	2	3
P vs Dd2^{Dd2}	–	0.35	0.010	0.010	0.006	0.40	0.80	>0.99
P vs Dd2$^{Dd2+F145I}$	0.010	–	–	0.12	0.22	0.024	0.071	0.024
**200 nM**	**3.8 ± 0.8**	**2.8 ± 0.6**	**18.4 ± 3.4**	**14.2 ± 2.4**	**12.1 ± 1.7**	**1.7 ± 0.2**	**3.2 ± 0.7**	**4.3 ± 1.1**
N	4	6	6	7	8	3	2	3
P vs Dd2^{Dd2}	–	0.26	0.010	0.006	0.016	0.40	0.53	>0.99
P vs Dd2$^{Dd2+F145I}$	0.010	–	–	0.17	0.23	0.024	0.071	0.024
**100 nM**	**4.3 ± 0.9**	**2.8 ± 0.5**	**15.4 ± 2.4**	**14.2 ± 2.4**	**10.1 ± 1.3**	**1.4 ± 0.1**	**4.2 ± 0.9**	**4.5 ± 1.1**
N	4	6	6	8	8	3	2	3
P vs Dd2^{Dd2}	–	0.26	0.010	0.004	0.038	0.057	0.53	>0.99
P vs Dd2$^{Dd2+F145I}$	0.010	–	–	0.47	0.12	0.024	0.071	0.024
**50 nM**	**4.1 ± 0.06**	**2.4 ± 0.5**	**17.5 ± 2.6**	**18.6 ± 2.9**	**11.9 ± 3.0**	**1.6 ± 0.4**	**2.6 ± 0.7**	**26.3 ± 13.7**
N	4	6	7	7	8	3	2	3
P vs Dd2^{Dd2}	–	0.038	0.005	0.010	0.010	0.057	0.13	0.057
P vs Dd2$^{Dd2+F145I}$	0.005	–	–	0.78	0.17	0.024	0.071	>0.99
**25 nM**	**8.5 ± 1.5**	**5.0 ± 1.0**	**18.2 ± 4.3**	**28.8 ± 5.9**	**36.1 ± 7.2**	**22.2 ± 6.1**	**20.4 ± 11.4**	**50.8 ± 18.3**
N	5	6	5	6	8	3	2	3
P vs Dd2^{Dd2}	–	0.13	0.016	0.009	0.008	0.38	0.14	0.036
P vs Dd2$^{Dd2+F145I}$	0.016	–	–	0.40	0.048	0.96	0.81	0.054
**12.5 nM**	**28.8 ± 5.3**	**44.7 ± 1.1**	**53.8 ± 5.4**	**38.6 ± 7.1**	**67.1 ± 7.7**	**58.6 ± 9.8**	**58.0 ± 24.7**	**75.9 ± 15.8**
N	4	6	7	6	4	3	2	3
P vs Dd2^{Dd2}	–	0.016	0.006	0.35	0.029	0.057	0.53	0.057
P vs Dd2$^{Dd2+F145I}$	0.006	–	–	0.35	0.15	0.95	>0.99	0.25
**6.25 nM**	**58.6 ± 6.5**	**77.6 ± 3.0**	**69.2 ± 3.2**	**54.2 ± 6.3**	**79.2 ± 5.1**	**78.3 ± 4.4**	**70.6 ± 5.1**	**89.0 ± 4.0**
N	4	5	7	6	8	3	2	3
P vs Dd2^{Dd2}	–	0.032	0.21	0.91	0.064	0.057	>0.99	0.057
P vs Dd2$^{Dd2+F145I}$	0.21	–	–	0.093	0.10	0.17	>0.99	0.033
**3.125 nM**	**79.0 ± 3.0**	**89.9 ± 2.2**	**82.9 ± 2.9**	**69.7 ± 5.1**	**87.4 ± 3.0**	**92.6 ± 12.4**	**99.6 ± 0.0**	**91.5 ± 3.1**
N	4	4	7	5	5	2	1	3
P vs Dd2^{Dd2}	–	0.057	0.39	0.11	0.19	0.53	–	0.11
P vs Dd2$^{Dd2+F145I}$	0.39	–	–	0.043	0.25	0.47	–	0.18

Piperaquine survival assay (PSA) values (nM) indicate the mean ± SEM, as determined in 2 to 8 independent assays performed in duplicate. Parasite survival is defined as the ratio of the parasitemias of the PPQ-treated to the no-drug control wells. This assay measures the survival of synchronous ring-stage parasites (0-6 hr post-invasion) exposed to PPQ for 72 hr, prior to measuring parasitemias by flow cytometry. N, number of independent assays. Statistical significance was determined via non-parametric Mann-Whitney *U* tests. *P* values are reported for comparisons with the parasite lines Dd2^{Dd2} and Dd2$^{Dd2+F145I}$.

*$P < 0.05$ **$P < 0.01$

Table S2 Mean IC_{50} and IC_{90} values of *pfcrt*-modified parasite lines.

	Dd2^{Dd2}	Dd2^{3D7}	Dd2$^{Dd2+F145I}$	Dd2$^{Dd2+F145I+F131C}$	Dd2$^{Dd2+F145I+F131C}$ (edited)	Dd2$^{Dd2+F145I+I347T}$	Dd2$^{Dd2+F145I+I347T}$ (edited)	Dd2$^{Dd2+F145I+C258W}$ (edited)
**PPQ IC_{50} (nM)**	**13.0 ± 1.3**	**11.4 ± 1.1**	**21.5 ± 1.6**	**12.3 ± 0.4**	**16.1 ± 1.7**	**15.4 ± 1.0**	**18.4 ± 1.4**	**11.1 ± 1.5**
N	7	7	6	6	7	3	3	3
P vs Dd2^{Dd2}	–	0.40	0.005	0.21	0.95	0.27	0.007	0.48
P vs Dd2$^{Dd2+F145I}$	0.005	–	–	0.002	0.073	0.024	0.26	0.024
**PPQ IC_{90} (nM)**	**23.7 ± 1.9**	**23.6 ± 3.5**	**5636 ± 690.9**	**3601 ± 236.6**	**2753 ± 225.9**	**28.1 ± 4.2**	**30.1 ± 3.3**	**22.6 ± 2.0**
P vs Dd2^{Dd2}	–	0.96	0.006	0.017	0.017	0.27	0.022	>0.99
P vs Dd2$^{Dd2+F145I}$	0.006	0.004	–	0.057	0.057	0.016	0.010	0.032
**CQ IC_{50} (nM)**	**94.1 ± 4.4**	**14.9 ± 1.6**	**30.0 ± 6.9**	**33.6 ± 4.5**	**30.1 ± 2.1**	**44.8 ± 6.0**	**42.7 ± 3.9**	**94.7 ± 5.8**
N	6	6	8	5	4	5	5	5
P vs Dd2^{Dd2}	–	0.002	0.001	0.004	0.010	0.004	0.004	0.792
P vs Dd2$^{Dd2+F145I}$	0.001	–	–	0.208	0.301	0.061	0.061	0.002
**CQ IC_{90} (nM)**	**172.2 ± 18.5**	**21.9 ± 3.0**	**63.1 ± 20.1**	**60.2 ± 10.4**	**65.4 ± 6.7**	**98.7 ± 8.7**	**103.7 ± 5.7**	**175.1 ± 22.7**
P vs Dd2^{Dd2}	–	0.002	0.015	0.002	0.010	0.015	0.002	0.75
P vs Dd2$^{Dd2+F145I}$	0.015	–	–	0.31	0.41	0.15	0.15	0.032
**md-CQ IC_{50} (nM)**	**572.7 ± 77.6**	**21.1 ± 2.8**	**124.6 ± 19.6**	**165.7 ± 17.0**	**180.8 ± 20.6**	**205.8 ± 39.9**	**192.4 ± 26.8**	**840.8 ± 95.7**
N	6	6	5	5	4	5	5	4
P vs Dd2^{Dd2}	–	0.002	0.004	0.004	0.004	0.004	0.004	0.083
P vs Dd2$^{Dd2+F145I}$	0.004	–	–	0.31	0.095	0.095	0.032	0.008
**md-CQ IC_{90} (nM)**	**1152 ± 143.2**	**347.9 ± 5.9**	**316.9 ± 69.9**	**397.4 ± 57.1**	**366.6 ± 41.5**	**600.2 ± 83.4**	**644.2 ± 68.3**	**1641 ± 86.4**
P vs Dd2^{Dd2}	–	0.002	0.010	0.004	0.010	0.004	0.004	0.052
P vs Dd2$^{Dd2+F145I}$	0.010	–	–	0.29	0.49	0.11	0.032	0.016
**md-ADQ IC_{50} (nM)**	**26.0 ± 4.8**	**11.7 ± 2.6**	**14.5 ± 2.8**	**18.6 ± 5.9**	**16.2 ± 2.9**	**21.8 ± 1.9**	**23.3 ± 0.6**	**24.1 ± 4.1**
N	5	5	5	4	5	5	4	4
P vs Dd2^{Dd2}	–	0.032	0.095	0.19	0.095	0.56	>0.99	0.75
P vs Dd2$^{Dd2+F145I}$	0.095	–	–	0.41	0.22	0.22	0.19	0.11
**md-ADQ IC_{90} (nM)**	**36.7 ± 7.3**	**16.9 ± 3.1**	**25.7 ± 7.2**	**32.9 ± 8.3**	**31.6 ± 6.1**	**38.9 ± 4.4**	**37.1 ± 4.4**	**36.9 ± 7.1**
P vs Dd2^{Dd2}	–	0.016	0.087	0.19	0.15	0.69	>0.99	0.73
P vs Dd2$^{Dd2+F145I}$	0.087	–	–	0.37	0.21	0.21	0.21	0.13
**QN IC_{50} (nM)**	**245.7 ± 56.5**	**427.7 ± 26.1**	**237.1 ± 37.4**	**220.6 ± 14.8**	**257.2 ± 48.9**	**217.7 ± 5.9**	**240.4 ± 20.1**	**257.2 ± 48.9**
N	5	5	5	4	4	6	6	**4**
P vs Dd2^{Dd2}	–	0.032	0.84	0.73	0.90	0.54	0.79	>0.99
P vs Dd2$^{Dd2+F145I}$	0.84	–	–	0.90	0.90	0.79	0.79	0.56
**QN IC_{90} (nM)**	**745.0 ± 113.2**	**923.2 ± 31.7**	**652.5 ± 75.4**	**709.7 ± 55.4**	**623.5 ± 45.7**	**637.0 ± 73.7**	**663.8 ± 34.3**	**n.d.**
P vs Dd2^{Dd2}	–	0.41	0.49	0.89	0.86	0.26	0.76	–
P vs Dd2$^{Dd2+F145I}$	0.49	–	–	0.49	0.23	0.76	0.91	–
**PND IC_{50} (nM)**	**5.8 ± 0.8**	**6.0 ± 1.0**	**6.0 ± 0.6**	**6.7 ± 1.1**	**6.4 ± 0.9**	**7.9 ± 0.9**	**7.2 ± 0.9**	**3.2 ± 0.6**
N	4	4	4	4	4	6	6	4
P vs Dd2^{Dd2}	–	0.89	0.69	0.69	0.69	0.11	0.48	0.11
P vs Dd2$^{Dd2+F145I}$	0.69	–	–	0.49	>0.99	0.25	0.54	0.029
**PND IC_{90} (nM)**	**9.4 ± 1.3**	**9.6 ± 1.3**	**11.4 ± 0.4**	**12.3 ± 0.9**	**12.3 ± 2.2**	**14.4 ± 2.4**	**9.4 ± 1.4**	**6.4 ± 1.6**
P vs Dd2^{Dd2}	–	>0.99	0.11	0.11	0.63	0.20	0.51	0.11
P vs Dd2$^{Dd2+F145I}$	0.11	–	–	0.69	0.49	0.35	0.41	0.057
**MFQ IC_{50} (nM)**	**7.1 ± 1.0**	**12.2 ± 1.1**	**6.0 ± 1.0**	**5.9 ± 1.0**	**6.4 ± 1.2**	**6.9 ± 0.2**	**5.8 ± 1.1**	**6.0 ± 0.72**
N	6	6	6	5	5	3	4	4
P vs Dd2^{Dd2}	–	0.015	0.48	0.54	0.79	0.55	0.61	0.91
P vs Dd2$^{Dd2+F145I}$	0.48	–	–	0.79	0.79	0.55	0.91	0.35
**MFQ IC_{90} (nM)**	**21.6 ± 3.2**	**31.7 ± 3.6**	**18.5 ± 1.6**	**20.0 ± 2.5**	**19.5 ± 2.5**	**19.8 ± 1.7**	**16.0 ± 1.5**	**14.9 ± 2.3**
P vs Dd2^{Dd2}	–	0.18	0.31	0.79	0.54	0.48	0.35	0.26
P vs Dd2$^{Dd2+F145I}$	0.31	–	–	0.33	0.54	0.48	0.39	0.17

IC_{50} and IC_{90} values (nM) are presented as the means ± SEM, as determined in 2 to 7 independent assays perfomed in duplicate. PPQ, piperaquine; CQ, chloroquine; md-CQ, monodesethyl-chloroquine; md-ADQ, monodesethyl-amodiaquine; QN, quinine; PND, pyronaridine; MFQ, mefloquine; N, number of assays. n.d., not determined. Statistical significance was determined via Mann Whitney *U* tests. *P* values are reported for comparisons with the parasite line Dd2^{Dd2} and Dd2$^{Dd2+F145I}$.

*$P < 0.05$	**$P < 0.01$	***$P < 0.001$

Table S3 Transport in proteoliposomes.

Isoform	PPQ			CQ		
	1-min uptake (pmol/mg)	n	*P* v. Dd2+F145I	1-min uptake (pmol/mg)	n	*P* v. Dd2+F145I
**Dd2**	2.3 ± 0.3	5	0.016	6.5 ± 0.3	6	0.0095
**Dd2+F145I**	13.6 ± 2.2	4	—	1.9 ± 0.2	4	—
**Dd2+F145I+F131C**	13.1 ± 2.5	4	>0.99	2.3 ± 0.4	5	0.41
**Dd2+F145I+I347T**	13.1 ± 2.6	4	>0.99	2.5 ± 0.3	4	0.20
**Dd2+F145I+C258W**	6.3 ± 0.4	3	0.06	3.9 ± 0.5	3	0.06
**3D7**	0.5 ± 0.5	5	—	1.2 ± 0.3	4	—

Transport kinetics for ^{3}H-PPQ and ^{3}H-CQ were determined with the listed PfCRT variants reconstituted into proteoliposomes. Data (mean ± SD of n = 3-6 experiments) for the 1-min uptake of ^{3}H-PPQ or ^{3}H-CQ (depicted in **Fig. 2D and 2E**) are shown for each variant tested.

**P* <0.05	***P* <0.01

Table S4 All salt bridges found for all PfCRT isoforms.

Amino acid pair	Dd2	Dd2+F145I	Dd2+F145I+F131C	Dd2+F145I+I347T	Dd2+F145I+C258W
ASP57/LYS53	43.58	74.33	60.09	68.34	77.96
GLU54/LYS53	15.88	0.73	0.9	5.79	1.3
GLU54/ARG392	21.6	61.45	29.66	23.2	43.21
GLU54/ARG400	5.96	20.04	7.82	0	1.36
ASP57/LYS56	22.9	3.79	7.16	13.45	2.2
ASP57/ARG400	15.45	0	0.03	3.36	0
GLU207/LYS80	3.06	3.6	12.45	1.5	0
ASP368/ARG81	0	0.3	7.26	5.36	14.95
ASP311/LYS85	45.04	36.15	32.99	64.68	61.09
ASP368/LYS85	5.99	7.52	21.54	3.79	15.41
ASP241/LYS116	17.21	1.2	6.62	0.57	3.7
ASP137/ARG231	66.31	0	0	0	0
GLU208/ARG150	18.64	7.22	26.83	16.74	6.49
GLU198/ARG374	0	0	0	0.9	14.51
GLU204/LYS200	67.64	61.95	36.22	48.17	60.85
GLU204/ARG374	0	20.57	2	0.9	0
GLU208/LYS270	13.52	21.74	19.11	13.68	37.62
GLU232/LYS236	53.73	0	0	0	0
GLU271/LYS270	6.36	11.55	7.39	10.72	10.05
GLU271/LYS307	8.06	4.63	13.52	0.7	0
GLU299/LYS307	14.48	9.39	1.66	15.31	10.59
ASP310/LYS307	13.91	1.76	5.19	0.03	2.43
ASP313/LYS317	25	54.96	0.03	0.4	0
ASP338/LYS339	36.05	0	0	0	0
ASP377/ARG374	55.19	34.25	41.51	69.11	22.34
GLU399/ARG392	60.49	64.08	41.81	57.92	36.09
GLU399/LYS402	1.9	3.53	17.64	2.73	15.01

Interaction lifetimes are given as a percentage of time where the interacting species are within 4 Å of each other. Values shown are for interactions that exist for ≥10% of simulation time for at least one isoform. Green indicates a higher percentage and red indicates a low percentage.

Table S5 (page 1) Averaged log_2 fold change of the baseline peptide levels in the variant PfCRT lines versus $Dd2^{Dd2+F145I}$.

Peptide	Mass Spec Mode	Hb chain	$Dd2^{Dd2}$ / $Dd2^{Dd2+F145I}$	$Dd2^{Dd2+F145I+F131C}$ / $Dd2^{Dd2+F145I}$	$Dd2^{Dd2+F145I+I347T}$ / $Dd2^{Dd2+F145I}$	$Dd2^{Dd2+F145I+C258W}$ / $Dd2^{Dd2+F145I}$
AHVD	AHVD_pos	Hbα	1.04	0.36	0.47	
AV;LG;GL;VA	AV;LG;GL;VA_pos	Hbβ	-5.42	0.44	2.29	-3.46
AVMGN	AVMGN_neg	Hbβ	-0.29	-0.05	-0.25	
DALT	DALT_pos	Hbα	-2.48	-0.72	-0.65	
DAVM	DAVM_pos	Hbβ				-3.46
DEVGG	DEVGG_pos	Hbβ	-10.92	0.78	1.30	-3.39
DGLAH	DGLAH_pos;DGLAH_neg	Hbβ				-0.76
DK	DK_pos	Hbα, Hbβ				-0.67
DKFLASV	DKFLASV_pos	Hbα				-4.22
DKL	DKL_neg	Hbβ	-1.51	-0.79	-2.37	-0.52
DLH;HLD	DLH;HLD_neg	Hbα	-11.85	0.50	1.14	
DLHA;AHLD	DLHA;AHLD_pos	Hbβ				-2.77
DLS	DLS_neg	Hbβ	-0.91	-0.37	-1.12	-2.98
DLS;LSD;SDL;SLD	DLS;LSD;SDL;SLD_neg; DLS;LSD;SDL;SLD_pos	Hbα				-0.81
DP	DP_neg	Hbα				-0.54
DPEN	DPEN_neg	Hbβ	0.54	-0.29	-0.95	-2.16
DPENF	DPENF_neg;DPENF_pos	Hbβ				-1.72
DPVN	DPVN_pos	Hbα	0.29	-0.63	-1.37	
DPVNF	DPVNF_neg	Hbα				0.02
ERM	ERM_pos	Hbα				-0.84
ESFGDLSTP	ESFGDLSTP_pos	Hbβ	-8.18	0.21	0.25	-1.39
EV;DL;LD	EV;DL;LD_pos	Hbβ				-1.90
EVG;GDL;STP;DAV;DGL	EVG;GDL;STP;DAV;DGL_pos	Hbβ				-1.14
EVGGEA	EVGGEA_pos	Hbβ				-3.59
FD	FD_pos	Hbα				-1.39
FLSF	FLSF_neg	Hbα	-0.24	-0.33	-0.49	
GA;AG	GA;AG_neg	Hbα, Hbβ				0.21
GAHAGEYGA	GAHAGEYGA_pos	Hbα				0.20
GEALGRLL	GEALGRLL_pos	Hbβ	-1.78	1.07	0.72	
GEYG;SFGD;FSDG	GEYG;SFGD;FSDG_pos	Hbα	-12.02	0.93	0.53	-0.59
GKVGAH;QVKGH	GKVGAH;QVKGH_pos;GKVGAH;QVKGH_neg	Hbα				-0.66
GLAH	GLAH_pos	Hbβ	-0.07	-0.07	-0.08	
GNPK	GNPK_neg	Hbβ	-0.11	-0.65	-0.71	
HFDLSHGSAQ;HVDDMPNALS	HFDLSHGSAQ;HVDDMPNALS_neg	Hbα	-4.40	0.94	1.62	-1.42
HG;GH; HG	HG;GH; HG_pos	Hbα, Hbβ	0.22	-0.54	-0.60	
HGKKV	HGKKV_neg	Hbα, Hbβ				-0.89
HKLRV	HKLRV_pos	Hbα				-1.91
HL;LH	HL;LH_pos	Hbβ				0.02
HLD	HLD_neg	Hbβ				-2.77
HLDNLKG	HLDNLKG_pos	Hbβ	-10.94	0.80	0.75	-2.59
HVDD	HVDD_pos	Hbα	-4.29	0.28	0.22	
KAHGKK	KAHGKK_pos	Hbβ				0.18
KEFT	KEFT_pos	Hbβ				-2.02
KFLASVST	KFLASVST_pos	Hbα	0.29	0.02	0.04	0.37
KGHGK;GHGKK	KGHGK;GHGKK_neg	Hbβ	0.96	0.41	0.43	
KKVADALT;TNVKAAWG;RVDPVNF	KKVADALT;TNVKAAWG;RVDPVNF_pos	Hbα	-2.09	0.80	0.65	
KLRVDPV	KLRVDPV_pos	Hbα				-0.09
KVNVDEV	KVNVDEV_pos	Hbβ				-3.34
LDK	LDK_neg	Hbα				-0.52
LE	LE_pos	Hbα				-0.19
LGR;GRL	LGR;GRL_neg	Hbβ				0.16
LH;HL	LH;HL_pos	Hbα	-9.78	0.83	0.49	
LK;KL	LK;KL_pos	Hbβ	-0.17	0.58	0.12	
LLGNVLVCVLAH	LLGNVLVCVLAH_neg	Hbβ				-2.78
LRVD	LRVD_neg	Hbα	-5.01	1.11	2.28	
LRVDPVN	LRVDPVN_pos	Hbα	0.05	-1.34	-1.24	-0.23
LS;VT;SL;TV	LS;VT;SL;TV_pos	Hbα				-0.09
LSHCLLV	LSHCLLV_pos	Hbα				-2.84
LSPAD	LSPAD_neg	Hbα	-0.19	-0.21	-0.11	
LSPADKTNVKAA	LSPADKTNVKAA_neg	Hbα				-3.59
NP	NP_neg	Hbβ				-4.42
NPKV	NPKV_pos	Hbβ	-10.87	0.29	0.40	
NPKVKAHGKK	NPKVKAHGKK_pos	Hbβ				-4.09
NVDE;DEVGG	NVDE;DEVGG_pos	Hbβ	-11.27	0.78	0.55	
NVDEVG	NVDEVG_pos	Hbβ	-0.07	-0.26	-0.24	
NVDEVGGEALG	NVDEVGGEALG_neg	Hbβ	0.00	-0.66	-0.35	0.19
NVKAA;AQVKG;LRVD	NVKAA;AQVKG;LRVD_neg	Hbα	-7.49	0.81	1.63	-0.23
PA	PA_pos	Hbα	-7.18	0.77	1.98	
PAD;PDA	PAD;PDA_pos	Hbα	-3.69	-1.51	-1.93	-0.09
PAE	PAE_pos	Hbα	-12.24	0.42	0.94	-3.42
PD;DP	PD;DP_pos; PD;DP_neg	Hbβ				-0.54
PDA	PDA_pos	Hbβ	-0.11	0.51	-0.04	0.02
PE	PE_neg	Hbβ	-1.09	-0.62	-0.79	-2.07
PEE	PEE_neg	Hbβ				-0.80
PEEK	PEEK_pos	Hbβ				-1.97
PEN	PEN_pos	Hbβ				-3.26
PENF	PENF_pos	Hbβ	-6.98	0.93	1.87	

Table S5 (page 2) Averaged log_2 fold change of the baseline peptide levels in the variant PfCRT lines versus $Dd2^{Dd2+F145I}$.

Peptide	Mass Spec Mode	Hb chain	$Dd2^{Dd2}$ / $Dd2^{Dd2+F145I}$	$Dd2^{Dd2+F145I+F131C}$ / $Dd2^{Dd2+F145I}$	$Dd2^{Dd2+F145I+I347T}$ / $Dd2^{Dd2+F145I}$	$Dd2^{Dd2+F145I+C258W}$ / $Dd2^{Dd2+F145I}$
PK	PK_pos	Hbβ	-4.73	0.83	1.70	-1.88
PKVK	PKVK_pos	Hbβ				-3.52
PN	PN_neg	Hbα	-10.24	0.53	1.48	
PN;NP	PN;NP_pos	Hbα, Hbβ	-0.98	-0.62	-0.61	
PNA	PNA_pos	Hbα				-4.42
PNALS	PNALS_pos	Hbα				-2.23
PPVQ	PPVQ_neg	Hbβ	-7.96	0.84	1.70	-1.43
PT;TP	PT;TP_pos	Hbα	-8.58	1.00	2.32	-2.88
PTT;DAL	PTT;DAL_pos; PTT;DAL_neg	Hbα, Hbβ				-3.71
PV	PV_pos	Hbα	-10.52	0.97	1.76	-2.38
PVN	PVN_pos	Hbα	-3.03	-1.21	-2.54	-4.52
PVNF	PVNF_neg	Hbβ	-7.46	1.34	2.28	-5.44
PVQ	PVQ_pos	Hbβ				-4.11
PVQA	PVQA_pos	Hbβ	-1.13	-0.43	-0.64	-3.96
PWT	PWT_pos	Hbβ	0.91	-1.63	-2.14	-2.88
PWTQ	PWTQ_pos	Hbβ				-2.84
QKVVA	QKVVA_pos	Hbβ	0.15	0.58	-1.27	
RF;FR	RF;FR_pos	Hbβ	-2.75	1.30	0.64	
SDLHA;HASLD	SDLHA;HASLD_pos	Hbα				-3.66
SFGD;FSDG	SFGD;FSDG_pos	Hbβ	1.97	0.35	0.35	
SFGDLSTP	SFGDLSTP_pos	Hbβ				-0.61
SFPTT	SFPTT_pos	Hbα	-0.19	-0.50	0.07	-3.59
STPDAVM;VDPENF	STPDAVM;VDPENF_neg	Hbβ				-1.48
TLAA	TLAA_neg	Hbα				-0.52
TNVK	TNVK_pos	Hbα				-0.19
TP	TP_neg	Hbβ				-3.44
TPAVH	TPAVH_pos	Hbα	-2.42	1.93	1.02	
TPAVH;KEFT	TPAVH;KEFT_pos	Hbα	0.08	-0.21	-0.30	
TPDA	TPDA_neg	Hbβ				-3.30
TPEE	TPE_pos	Hbα				-3.26
TPEEK;LSTPDA	TPEEK;LSTPDA_pos	Hbβ				-2.20
TSKY	TSKY_neg	Hbα	0.48	0.30	-0.53	
TYFP	TYFP_pos	Hbα	-1.91	-0.52	-0.76	
V	V_pos	Hbα, Hbβ	1.06	-0.06	-0.23	
VAGVANA	VAGVANA_neg	Hbβ	0.64	0.46	0.58	
VAGVANAL	VAGVANAL_neg	Hbβ	0.47	0.36	0.60	
VAHV	VAHV_pos	Hbα	-9.43	-0.16	0.05	
VAHVDDMP	VAHVDDMP_pos	Hbα	-0.39	0.37	0.11	
VC;CV	VC;CV_neg	Hbβ	-9.00	0.70	0.64	-0.42
VD	VD_pos; VD_neg	Hbα, Hbβ	-0.17	0.12	-0.27	-1.98
VDD	VDD_neg; VDD_pos	Hbα	-3.43	0.01	1.21	
VDE;DEV	VDE;DEV_pos	Hbβ	-2.24	2.00	1.67	-1.03
VDEVG	VDEVG_pos	Hbβ				-2.18
VDEVGGEALG	VDEVGGEALG_neg	Hbβ	0.72	0.40	0.44	
VDPENF	VDPENF_pos	Hbβ	-0.80	-0.91	-1.61	
VDPVN	VDPVN_neg	Hbα				-2.44
VG	VG_neg	Hbα	-0.18	0.33	0.11	-3.62
VG;GV	VG;GV_neg;VG;GV_pos	Hbβ				-3.39
VHAS	VHAS_pos	Hbα	-0.81	-0.68	-1.17	
VHASL	VHASL_neg	Hbα				-3.39
VHL;LHV	VHL;LHV_pos	Hbβ				0.30
VLSP	VLSP_pos	Hbα	-1.44	-0.89	-1.87	
VNVDEVG;EVGGEALG	VNVDEVG;EVGGEALG_neg	Hbβ				0.17
VT;LS	VT;LS_pos	Hbβ	-2.16	0.69	-0.29	
VV	VV_pos	Hbβ	-0.04	0.15	-0.07	
VVYP	VVYP_neg	Hbβ				-0.79
YH	YH_pos	Hbβ				-0.61

134 peptides were detected in either positive or negative mode. Blank spaces denote missing or undetectable values.

Table S6 (page 1) List of peptides showing significantly different levels for Dd2^{Dd2}, Dd2$^{Dd2+F145I+F131C}$, Dd2$^{Dd2+F145I+I347T}$, and Dd2$^{Dd2+F145I+C258W}$ compared to Dd2$^{Dd2+F145I}$.

Peptide	IEP	pH at 5.5	pH at 7.4	*P*-value	Mean of log2-transformed peak areas of Dd2$^{Dd2+F145I}$	Mean of log2-transformed peak areas of Dd2^{Dd2}	Difference in log_2-transformed mean peak areas	SE of difference
AHVD	4.78	-0.4	-1	0.048	14.3	15.3	1.0	0.36
AV;LG;GL;VA	5.98; 5.98; 5.98; 5.98	0; 0; 0; 0	0; 0; 0; 0	0.009	11.0	5.6	-5.4	1.05
DGLAH	4.78	-0.4	-1	0.001	12.2	1.3	-10.9	0.68
DLHA;AHLD	4.78; 4.78	-0.4; 0.4	-1; -1	0.002	14.9	3.1	-11.9	0.80
EV;DL;LD	3.64; 3.37; 3.37	-0.9; -1; -1	-1;-1; -1	0.0002	14.7	6.5	-8.2	0.63
GKVGAH;QVKGH	9.37	3.2	2	0.0001	17.1	5.1	-12.0	0.57
HG;GH	7.61	1.7	0	0.003	12.3	7.9	-4.4	0.30
HVDD	3.93	-1.4	-2	0.00002	15.0	4.1	-10.9	0.40
KKVADALT;TNVKAAWG;RVDPVNF	8.76; 9.07; 6.5	1; 1; 0	0.9; 1; 0	0.011	15.0	15.9	15.0	0.96
LH;HL	7.37; 7.37	1.2; 1.2	0; 0	0.00003	12.8	3.1	-9.8	0.47
LRVDPVN	6.5	0	0	0.011	10.6	5.6	-5.0	0.85
NPKVKAHGKK	9.66	4.6	3.9	0.001	11.8	0.9	-10.9	0.78
NVDE;DEVGG	3.29; 3.29	-1.9; -1.9	-2; -2	0.017	14.2	10.4	-3.8	0.86
NVDEVG	3.29	-1.9	-2	0.0001	16.1	4.8	-11.3	0.36
PA	5.98	0	0	0.001	11.7	4.2	-7.5	0.49
PAD;PDA	3.17; 3.17	-2; -2	-2; -2	0.005	10.1	3.0	-7.2	0.93
PD;DP	3.37; 3.37	-2; -2	-2; -2	0.00001	15.1	2.9	-0.1	0.40
PK	9.07	1	1	0.001	12.5	5.5	-7.0	0.76
PKVK	9.37	2	1.9	0.004	11.3	6.6	-4.7	0.76
PN;NP	5.98; 5.98	0; 0	0; 0	0.0004	12.1	1.9	-10.2	0.63
PT;TP	5.98; 5.98	0; 0	0; 0	0.0001	12.9	4.9	-8.0	0.54
PTT;DAL	5.98; 3.37	0; -1	0; -1	0.008	12.2	3.6	-8.6	0.94
PVN	5.98	0	0	0.0001	13.5	3.0	-10.5	0.64
PVNF	5.98	0	0	0.005	14.7	11.7	-3.0	0.54
PVQ	5.98	0	0	0.001	11.0	3.5	-7.5	0.70
VAHVDDMP	3.93	-1.4	-2	0.002	14.0	4.6	-9.4	0.89
VD	3.37	-1	-1	0.005	11.6	2.6	-9.0	0.77

Peptide	IEP	pH at 5.5	pH at 7.4	*P*-value	Mean of log2-transformed peak areas of Dd2$^{Dd2+F145I}$	Mean of log2-transformed peak areas of Dd2$^{Dd2+F145I+F131C}$	Difference in log_2-transformed mean peak areas	SE of difference
NVDE;DEVGG	3.29	-1.9	-2	0.037	14.2	16.0	1.8	0.57

Peptide	IEP	pH at 5.5	pH at 7.4	*P*-value	Mean of log2-transformed peak areas of Dd2$^{Dd2+F145I}$	Mean of log2-transformed peak areas of Dd2$^{Dd2+F145I+I347T}$	Difference in log_2-transformed mean peak areas	SE of difference
DGLAH	4.78	-0.4	-1	0.025	12.2	13.5	1.3	0.34
DLHA;AHLD	4.78; 4.78	-0.4; 0.4	-1; -1	0.029	14.9	16.1	1.1	0.29
HG;GH	7.37; 7.37	0.6; 0.6	0; 0	0.023	12.3	13.9	1.6	0.45
PA	5.98	0	0	0.048	11.7	13.3	1.6	0.47
PAD;PDA	3.17	-2	-2	0.043	10.1	12.1	2.0	0.67
PN;NP	5.98	0	0	0.026	12.1	13.6	1.5	0.35
PT;TP	5.98	0	0	0.022	12.9	14.6	1.7	0.45
PVN	5.98	0	0	0.040	13.5	15.3	1.8	0.55

Table S6 (page 2) List of peptides showing significantly different levels for Dd2^{Dd2}, Dd2$^{Dd2+F145I+F131C}$, Dd2$^{Dd2+F145I+I347T}$, and Dd2$^{Dd2+F145I+C258W}$ compared to Dd2$^{Dd2+F145I}$.

Peptide	IEP	pH at 5.5	pH at 7.4	*P*-value	Mean of log2-transformed peak areas of Dd2$^{Dd2+F145I}$	Mean of log2-transformed peak areas of Dd2$^{Dd2+F145I+C258W}$	Difference in log_2-transformed mean peak areas	SE of difference
AV;LG;GL;VA	5.98; 5.98; 5.98; 5.98	0; 0; 0; 0	0; 0; 0; 0	0.002	13.6	10.2	-3.5	0.51
DEVGG	3.29	-1.9	-2	0.008	14.5	11.1	-3.4	0.61
DKFLASV	6.34	0	0	0.001	15.5	11.3	-4.2	0.35
DLH	4.77	-0.8	-2	0.025	15.8	13.1	-2.8	0.70
DMPNA	3.37	-1	-1	0.031	13.5	12.1	-1.4	0.42
EVGGEA	3.02	-1.8	-2	0.040	12.5	8.9	-3.6	1.00
HLD	4.78	-0.4	-1	0.025	15.8	13.1	-2.8	0.70
KVNVDEV	4.19	-0.9	-1	0.046	13.7	10.4	-3.3	1.13
LSHCLLV	6.59	0.6	-0.4	0.026	13.9	11.1	-2.8	0.72
LSPAD	3.37	-1	-1	0.013	13.6	10.0	-3.6	0.74
NP	5.98	0	0	0.004	15.3	10.9	-4.4	0.69
NPKV	9.07	1	1	0.028	17.4	13.3	-4.1	0.88
PAE	3.64	-0.9	-1	0.021	14.2	10.8	-3.4	0.81
PEEK	4.44	-0.8	-1	0.034	17.6	15.7	-2.0	0.51
PEN	3.64	-0.9	-1	0.020	15.2	12.0	-3.3	0.77
PENF	3.64	-0.9	-1	0.027	14.7	12.8	-1.9	0.44
PN	5.98	0	0	0.004	15.3	10.9	-4.4	0.69
PTT;DAL	5.98; 3.37	0; -1	0; -1	0.019	12.7	9.0	-3.7	0.85
PVN	5.98	0	0	0.044	16.9	12.4	-4.5	1.18
PVNF	5.98	0	0	0.001	15.4	10.0	-5.4	0.69
PVQ	5.98	0	0	0.012	13.6	9.5	-4.1	0.92
PVQA	5.98	0	0	0.009	15.7	11.8	-4.0	0.77
PWTQ	5.98	0	0	0.026	13.9	11.1	-2.8	0.72
QKVVA	9.07	1	1	0.005	13.3	9.6	-3.7	0.66
TP	5.98	0	0	0.039	14.8	11.3	-3.4	0.92
TPAVH	7.37	0.6	0	0.036	18.1	14.8	-3.3	0.81
TPDAV	3.37	-1	-1	0.013	13.6	10.0	-3.6	0.74
TPE	3.64	-0.9	-1	0.020	15.2	12.0	-3.3	0.77
VG	5.98	0	0	0.002	13.2	9.5	-3.6	0.44
VG;GV	5.98; 5.98	0; 0	0; 0	0.012	14.5	11.1	-3.4	0.46
VHAS	7.37	0.6	0	0.012	14.5	11.1	-3.4	0.46

Data were obtained from three independent experiments with technical triplicates. For each peptide, the isoelectric point (IEP), pH at 5.5, and pH 7.4 are displayed. *P* values were calculated from unpaired *t* tests. Gray indicates peptides that were detected in more than one line.

Table S7 List of oligonucleotides used in this study.

Name	Nucleotide Sequence (5'-3')	Description	Lab name	Purpose
p1	CCCTTGTCGACCTTAACAGATGGCTC	*pfcrt* exon 2 SalI Fwd	p3519	Sequencing primer for *pfcrt*.
p2	TCAAACATGACAAGGGAAATAGT	*pfcrt* exon 5 Rev	p2427	
p3	CTCGAGatggttggttcgctaaactgc	hDHFR XhoI Fwd	p3315	Integration PCR #1. 2.5 kb yes/no. integration at 3' end Sequences exons 2-3.
p4	TTGACCCTTATATATTCCACCCA	*pfcrt* 3' UTR	p3403	
p5	cttgggCCCAAGTTGTACTGCTTCTAAGC	*pfcrt* 5' UTR (-494-517) ApaI Fwd	p3404	Integration PCR #2. 1.2 kb checks integration at 5' end (1.4 kb if unedited due to additional intron 2).
p6	cttatcgatAAGCAGAAGAACATATTAATAG GAATACTTAATTG	*pfcrt* exon 3 ClaI Rev	p3265	
p7	GACCTTAACAGATGGCTCAC	*pfcrt* exon 2 EcoRI Fwd	p3264	Integration PCR #3 primer (along with p6). 0.4 kb (0.6 kb if unedited due to additional intron 2). Sequences exons 2-3.
p8	aaccatggatTTATTGTGTAATAATTGAATCGACG	pfcrt exon 13 Rev	p1640	Integration PCR #4 primer (along with p5). 2 kb. Sequence entire edited locus.
p9	agccGGTGATGTTGTAAgAGAACCAAGATTATTAG	PfCRT F131C SDM Fwd	p7068	PfCRT F131C SDM
p10	CTAATAATCTTGGTTCTcTTACAACATCACCggct	PfCRT F131C SDM Rev	p7069	
p11	CCTGTTCAGTCATTTTGGCCaTCATAGGTCTTACA AGAACTAC	PfCRT F145I SDM Fwd	p6106	PfCRT F145I SDM
p12	GTAGTTCTTGTAAGACCTATGAtGGCCAAAATGAC TGAACAGG	PfCRT F145I SDM Rev	p6107	
p13	ctttttccaattgttcacttcttgGcttatattac ctgtatacaccctt	PfCRT C258W SDM Fwd	p8550	PfCRT C258W SDM
p14	aagggtgtatacaggtaatataagCcaagaagtga acaattggaaaaag	PfCRT C258W SDM Rev	p8551	
p15	aaattttctaccatgacatatactaCtgttagttg tatacaaggtccagca	PfCRT I347T SDM Fwd	p7374	PfCRT I347T SDM
p16	tgctggaccttgtatacaactaacaGtagtatatg tcatggtagaaaattt	PfCRT I347T SDM Rev	p7375	
p17	GAAGCTTTAATTTACAATTTTgTGCTATATCCATG TTAGATGCC	PfCRT F131C SDM Fwd	p8136	F131C SDM on *pfcrt* sequence codon optimized for S. *cerevisiae.*
p18	GGCATCTAACATGGATATAGCAcAAAATTGTAAAT TAAAGCTTC	PfCRT F131C SDM Rev	p8137	
p19	GCAGCGTCATCTTGGCCaTCATCGGTCTTACCAGA AC	PfCRT F145I SDM Fwd	p7698	F145I SDM on *pfcrt* sequence codon optimized for S. *cerevisiae.*
p20	GTTCTGGTAAGACCGATGAtGGCCAAGATGACGCT GC	PfCRT F145I SDM Rev	p7699	
p21	GTTAGCTTCTTCCAACTGTTCACTTCATGgTTAAT CCTGCCAGTTTACACACTACCATTC	PfCRT C258W SDM Fwd	p8821	C258W SDM on *pfcrt* sequence codon optimized for S. *cerevisiae.*
p22	GAATGGTAGTGTGTAAACTGGCAGGATTAAcCATG AAGTGAACAGTTGGAAGAAGCTAAC	PfCRT C258W SDM Rev	p8822	
p23	CTCCACCATGACTTACACTAcTGTGAGTTGCATCC AGGGGC	PfCRT I347T SDM Fwd	p8138	I347T SDM on *pfcrt* sequence codon optimized for S. *cerevisiae.*
p24	GCCCCTGGATGCAACTCACAgTAGTGTAAGTCATG GTGGAG	PfCRT I347T SDM Rev	p8139	
p25	CCGCGACTAGTGAGCTCGTCGAC	pFastBac Sequencing	p8157	pFastBac constructs sequencing primer

Fwd, forward primer; Rev, reverse; SDM, site-directed mutagenesis; UTR, untranslated region.

SUPPLEMENTARY REFERENCES

1. Straimer J, Lee MC, Lee AH, Zeitler B, Williams AE, Pearl JR, Zhang L, Rebar EJ, Gregory PD, Llinas M, Urnov FD, Fidock DA. 2012. Site-specific genome editing in *Plasmodium falciparum* using engineered zinc-finger nucleases. Nat Methods 9:993-8.
2. Kim J, Tan YZ, Wicht KJ, Erramilli SK, Dhingra SK, Okombo J, Vendome J, Hagenah LM, Giacometti SI, Warren AL, Nosol K, Roepe PD, Potter CS, Carragher B, Kossiakoff AA, Quick M, Fidock DA, Mancia F. 2019. Structure and drug resistance of the *Plasmodium falciparum* transporter PfCRT. Nature 576:315-320.
3. Willems A, Kalaw A, Ecer A, Kotwal A, Roepe LD, Roepe PD. 2023. Structures of *Plasmodium falciparum* Chloroquine Resistance Transporter (PfCRT) isoforms and their interactions with chloroquine. Biochemistry 62:1093-1110.
